# Metamaterial sensor based on rectangular enclosed adjacent triple circle split ring resonator with good quality factor for microwave sensing application

**DOI:** 10.1038/s41598-022-10729-4

**Published:** 2022-04-26

**Authors:** Md. Rashedul Islam, Mohammad Tariqul Islam, M. Salaheldeen M., Badariah Bais, Sami H. A. Almalki, Haitham Alsaif, Md. Shabiul Islam

**Affiliations:** 1 Department of Electrical, Electronic and Systems Engineering, Faculty of Engineering and Built Environment, Universiti Kebangsaan Malaysia, 43600 Bangi, Selangor Malaysia; 2 Department of Electrical Engineering, Faculty of Energy Engineering, Aswan University, Aswan, 81528 Egypt; 3 Department of Electrical Engineering, College of Engineering, Taif University, P.O. Box 11099, Taif, 21944 Kingdom of Saudi Arabia; 4 Department of Electrical Engineering, College of Engineering, University of Ha’il, Ha’il, 81481 Saudi Arabia; 5 Faculty of Engineering, Multimedia University, 63100 Cyberjaya, Selangor Malaysia

**Keywords:** Engineering, Materials science, Physics

## Abstract

In this article, a novel shaped metamaterial sensor is presented for the recognition of various oils, fluids, and chemicals using microwave frequency. The performance of the designed sensor structure has been studied both theoretically and experimentally, and it works well. A new sample holder for convenient operation is created and located just behind the designed structure. The results of this study performed better than those of prior liquids sensing studies. Various designs were explored using the Genetic Algorithm (GA), and it is embedded in the Computer Simulation Technology (CST) microwave studio, to optimize the optimal dimensions of the resonator. The suggested metamaterial sensor has a good-quality factor and sensitivity in both frequency shifting and amplitude changing. The resonance frequency shifted to 100 MHz between olive and corn oils, 70 MHz between sunflower and palm oils, 80 MHz between clean and waste brake fluids, and 90 MHz between benzene and carbon-tetrachloride chemicals. The quality factor of the sensor is 135, sensitivity is 0.56, and the figure of merit is 76 which expresses its efficient performance. Furthermore, the proposed sensor can sensitively distinguish different liquids by using the frequency shifting property. The study was carried out in three stages: dielectric constant (DK) measurement with the N1500A dielectric measurement kit, simulation of the structure, and experimental test study with the vector network analyzer. Since the recommended sensor has high sensitivity, good quality factor, and excellent performance, hence it can be used in chemical, oil, and microfluidic industries for detecting various liquid samples.

## Introduction

Metamaterials (MTMs) are artificial materials that are utilized to control and manipulate electromagnetic (EM) waves, sound, and other physical phenomena in unusual ways other than regular materials by utilizing the structure's natural properties. Following the experimental discovery of negative permittivity and permeability properties of metamaterials, scientists proposed various uses^1,2^. In 2006, the "Invisibility Cloak" was sparked, and more cloaking research has been carried out since then. Energy harvesting applications have been created using microwave metamaterial absorbers. There were also several metamaterial-inspired antenna applications designed for specific uses. In addition to these investigations, metamaterial sensor research has been produced for a variety of applications and objectives. Biosensors^3–5^, absorbers^6–8^, antennas^9–12^, energy harvesting^13,14^, microwave sensors^15,16^, and microwave lenses are some of the uses of MTMs. The design of the MTM-based sensor has gained a lot of interest from researchers in the field of microwave sensors^17,18^. Using the finite element method (FEM) and finite integration technique (FIT) simulation tools, different attractive designs and architectures can be made possible to improve the MTMs sensor’s sensitivity. The information of permittivity can be broadly used for emerging sensors in microwave frequency. MTM sensor gives the different resonant frequencies due to capacitance change since the capacitance affects the dielectric constant of the sample in the sensing region.

An oval-shaped MTM sensor is described^19^ for measuring glucose intensity in an aqueous mixture. The sensitivity of the sensor was calculated to be 0.037 GHz per 30 mg/dL solution of glucose. A labyrinth-shaped MTM is illustrated^20^, for sensing the oil quality. The operating frequency is 2–6 GHz, frequency shifted to 40 MHz and − 1.5 dB amplitude changed. A microfluidic sensor is presented^21^ for the sensing of ethanol chemicals. Ethanol concentration for this sensor is 0–100%, where the resonant frequency shifted from 10.42 to 11.46 GHz. Double negative (DNG) metamaterial based on V loaded CSSRR resonator is presented^22^, for the applications of radar and Wi-Fi. The size and effective medium ratio (EMR) of the study are 8 × 8 mm^2^, and 13.11. A novel MTM based hypersensitized sensor is presented^23^, for detecting different liquids. The sensor works within the 1–8 GHz frequency and the frequency shifted to 63 MHz with a − 0.9 dB magnitude change for clean and waste samples. Absorbers based on graphene are discussed^24^ for various applications. Absorber sensors are one of them, and they may be used in biosensors to detect hemoglobin and urine biomolecules, moisture detection, and medical applications by utilizing graphene's tuning characteristics.^25^, a graphene-based biosensor with an SRR metasurface is discussed for sensing hemoglobin or urine biomolecules. This graphene-based biosensor's high sensitivity tunable properties could be useful in medical applications for detecting urine and hemoglobin biomolecules. In^26^, a highly sensitive surface plasmon resonance (SPR)-based sensor with a circular polycrystalline air holes structure is proposed for alcohol detection, with higher performance. In this study, various alcohol series such as ethanol, methanol, butanol, propanol, pentanol, and phenol are used and showed the sensing performance. Another MTM based sensor incorporating a transmission line is demonstrated^27^, for the detection of pure and adulterated diesel. The operating frequency range was 8–12 GHz, the amplitude change was − 2.5 dB, and the frequency shifted to 60 MHz. An oval wing MTM is presented^28^, for chemical recognition. The frequency shifted to 120 MHz with a magnitude change of − 3.2 dB in the study. A Chiral MTM is presented^29^, for the sensing of microfluidic and fuel contamination. The resonance frequency is shifted 60 MHz with a − 8.50 dB amplitude change for the pure and 5% mixing gasoline samples. Split ring resonators are the most prevalent metamaterial resonance structure, and they can be merged with either a microstrip structure or a transmission line to measure the dielectric characteristics of liquids^30–32^. A 60 MHz resonance frequency shift MTM sensor was demonstrated^33^ for measuring the liquid's dielectric characteristics. The sensitivity of this structure is high, but the quality factor is moderate. For biological applications in real-time glucose detection, an active SRR is presented^34^. The structure's operating frequency is 1.156 GHz, and its Q-factor and sensitivity are also high. A novel microwave sensor is demonstrated^35^ for vanadium electrolyte sensing, where its resolution is very high. The sensor is not in direct contact with the electrolyte in this approach, hence temperature and displacement dependencies are reduced. Furthermore, because of the little maintenance required as a result of the contactless measurement, this low-cost design provides a long-lasting sensing platform.

In^36^ demonstrates the use of an MTM sensor to detect various oils. For dirty and clean transformer oils, the resonance frequency was altered to 70 MHz, while for olive oil and corn oil it was shifted to 50 MHz. A portable sensor with high efficiency is described^37^, for detecting branded and unbranded fuel samples. The resonance frequency shifted 72 MHz with a reflection magnitude change of − 4 dB for branded and unbranded diesel, whereas the frequency shifted 12 MHz with a reflection magnitude change of − 6 dB for branded and unbranded gasoline. A transmission line-based MTM is presented^38^, for defining original and adulterated gasoline samples. This sensor was able to discriminate between genuine and adulterated diesel samples with a 50 MHz frequency shift. According to a review of the literature, metamaterial-based sensors can be used in a wide frequency range^36,39^ and for a variety of materials, including solid dielectrics, liquids, gases, and biomolecules^40–42^. A sensitive MTM is expressed^43^, for the distinction between original and adulterated fuel samples. The resonance frequency is shifted 100 MHz with the reflection magnitude change of − 10 dB. Three rhombus MTM sensor is discussed^44^ for fuel sensing, but the sensitivity and quality factor are low in this study. In^45^, a meander line metamaterial-based sensor for polypropylene detection is demonstrated. The overall performance of this sensor was moderate. MTM absorber-inspired sensor is presented^46^, for detecting the liquid chemicals changing on the electrical characteristics. The quality factor and the sensitivity of the sensor are not sufficient. An omega shaped sensor is explained^47^, for industrial applications. The sensor works within 8–12 GHz frequency, 70 MHz frequency shifted for clean and waste transformer oils, and the Q-factor is moderate. Another MTM sensor is described^48^ to detect the liquid chemicals. The quality factor and sensitivity are moderate in the study.

In this study, a new MTM sensor based on adjacent triple circle SRR shaped with a quality factor is conceived and analyzed. We have investigated the width of the resonator, the gap of the split, radius of the circle, sample holder thickness, and substrate size for the recommended MTM sensor. Various samples have been detected and gained high sensitivity and good quality factor. The proposed sensor can identify various types of liquids such as oils, fluids, chemicals, adulterated mixtures with quality factor, high sensitivity, superior accuracy, and high figure of merits which are the extra features of the sensor. We have designed a novel sample holder to show the superior accuracy of the sensor. Also showed a better performance of the proposed sensor than the other reported MTM sensor (in Table 2). The quality factor, sensitivity, and figure of merits of the sensor are 135, 0.56, and 76 respectively. The reflection coefficient parameter which is directly affected by the dielectric constant of the samples placed in the sensor layer is used for analysis and recognition in this study. The organization of this article is as follows: design and analysis of the structure are presented in “Design and analysis of the structure”. Parametric analysis of the metamaterial sensor is described in “Parametric analysis of the metamaterial sensor”. Results and discussion are presented in “Results and discussions”, and the conclusion is presented in “Conclusion”.

## Design and analysis of the structure

**Figure 1:**
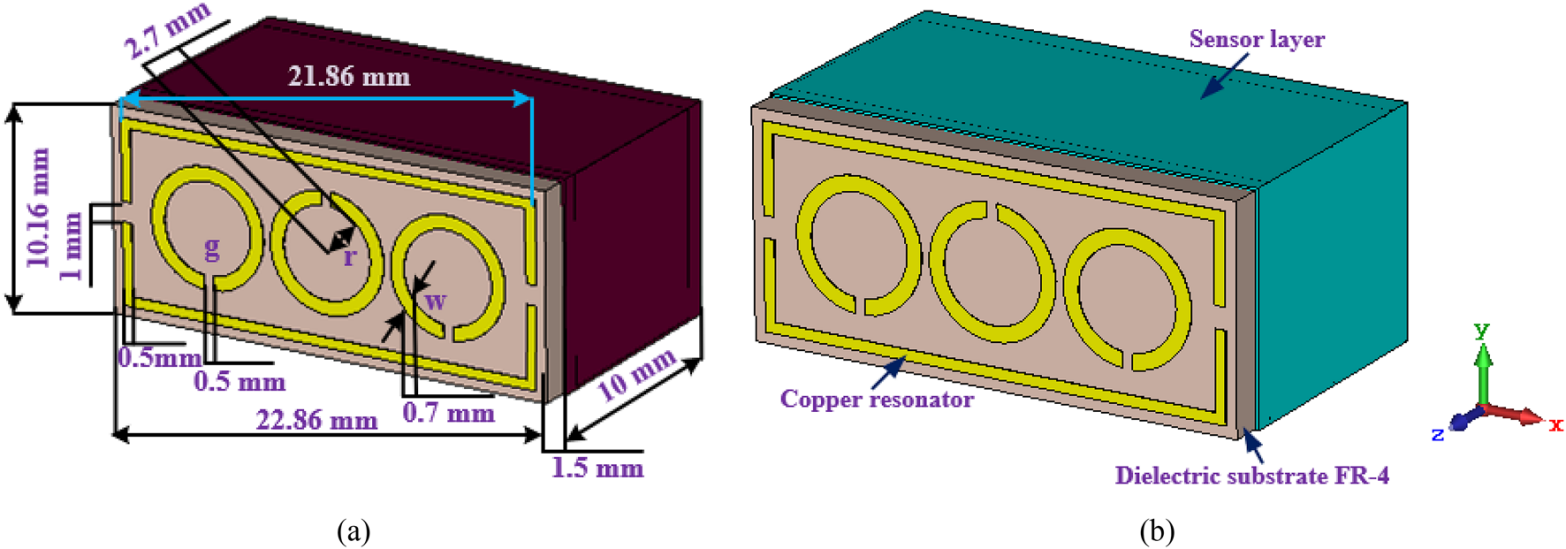
(**a**) Main construction with sizes, (**b**) perspective position of the MTM-based sensor. (CST STUDIO SUITE 2019, https://www.3ds.com/products-services/simulia/products/cst-studio-suite)^50^.

Figure 1a,b indicates the unit cell dimension and perspective position; it is well-suited for the waveguide (X-band). The whole length and width of the MTM sensor are 22.86 and 10.16 mm respectively, which is compatible with the X-band waveguide. The substrate material is flame retardant (FR-4) and the resonator is copper. On an FR-4 substrate with a thickness of 1.5 mm, a dielectric constant of 4.4, and a loss tangent of 0.02 the design is started. On both sides of the substrate, there were copper layers with a thickness of 0.035 mm. The sample holder is made of the polypropylene layer having a thickness of 1.5 mm, DK is 2.4, and the loss tangent is 0.004. The parametric analysis and the Genetic Algorithm (GA) approach were used to determine the required dimensions of the triple circle split SRR and the overall proposed structure. The GA approach was a built-in function inside the CST software^49^, which can be used to obtain the optimum result. A stochastic exploration technique acting on the tenets of pure genetic systems is a Genetic Algorithm. It operates a search to deliver the best possible result to an optimization issue for the condition function.

**Figure 2:**
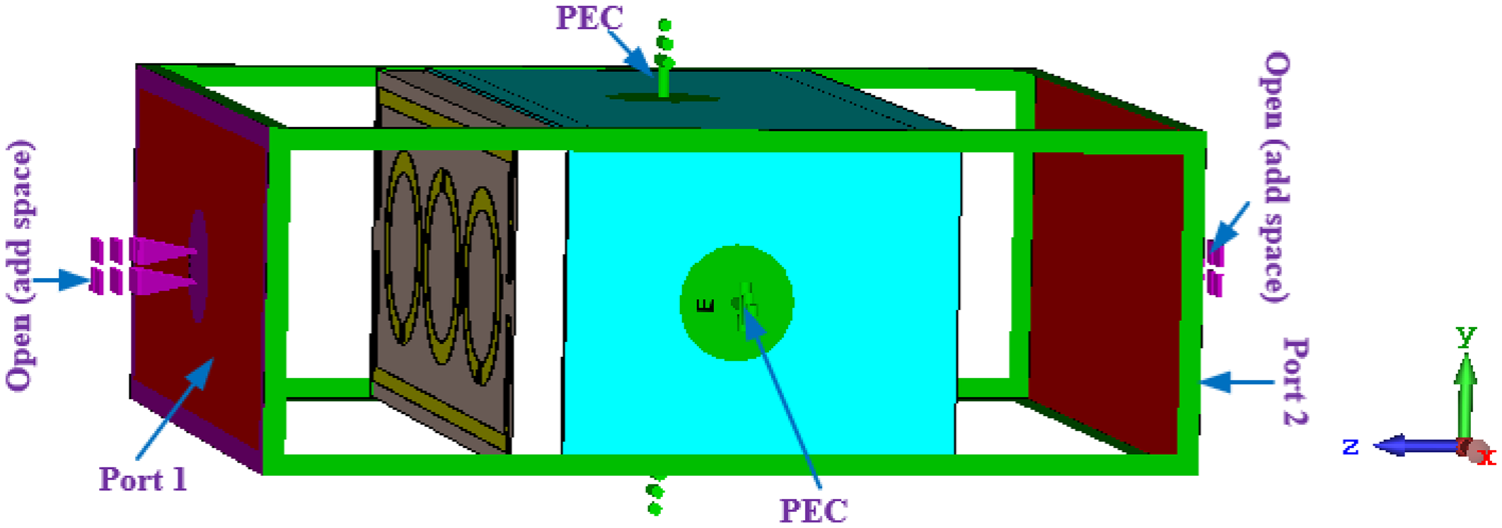
Design of MTM-based sensor: PEC-PEC open add spaces boundary conditions.

The goal of achieving waveguide measurements in the simulation procedure for the designed structure with the effective size is to apply different boundary conditions. Because of the side-wall waveguide's metallic composition, it is appropriate to take the boundary requirements into justification, containing free space, periodic distribution, perfect electric conductor (PEC)/perfect magnetic conductor (PMC), and PEC. In the z-axis, the normal incident electromagnetic wave is used, whereas in the x- and y-axes, the perfectly electrified boundary condition is applied as shown in Fig. 2.

**Figure 3:**
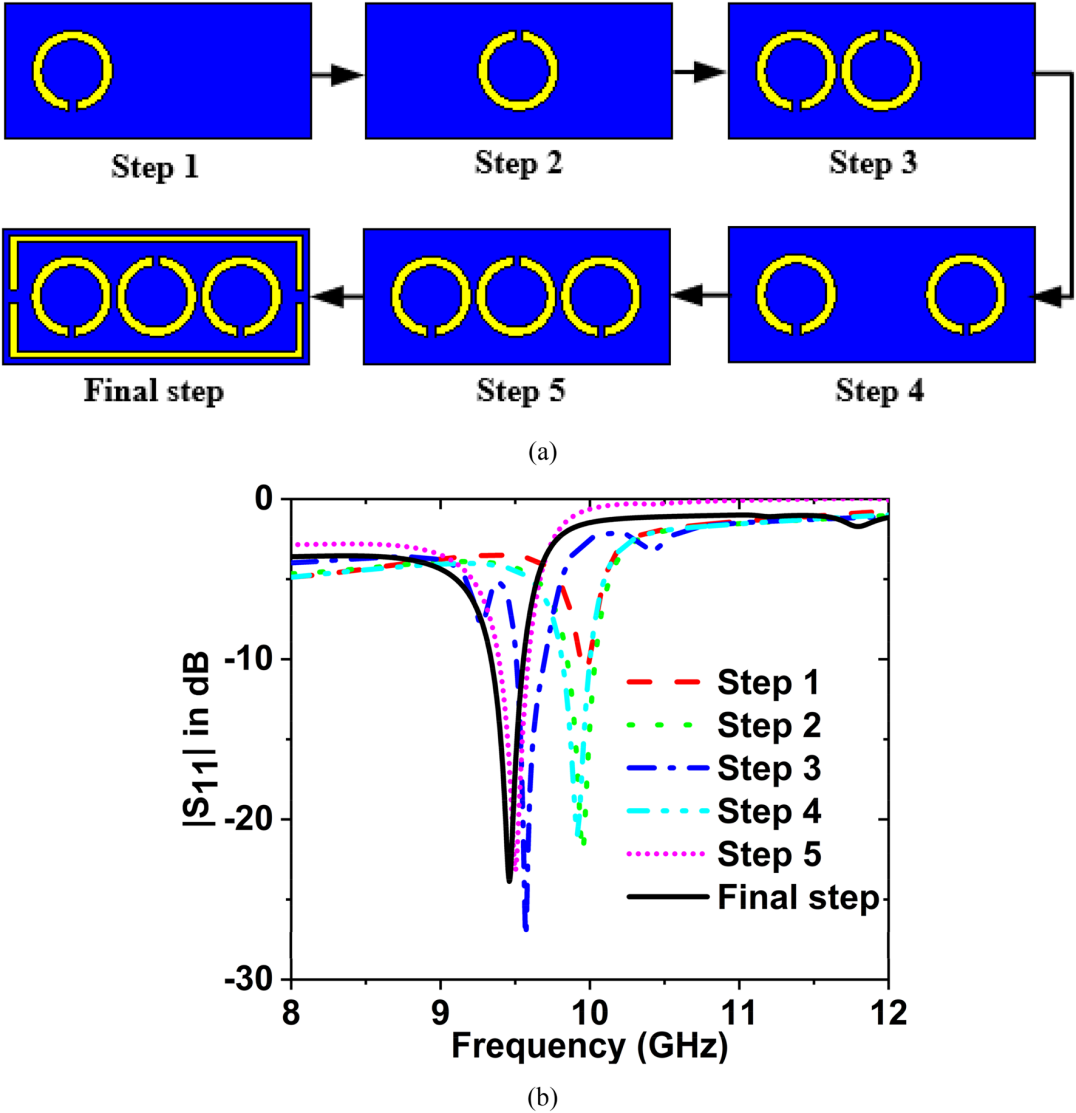
(**a**) Design procedure (**b**) Reflection coefficient (${S}_{11}$) for various design steps.

The design procedure of the suggested metamaterial sensor is depicted in Fig. 3a. The reflection coefficient (${S}_{11}$) for the various resonator designs of the MTM sensor are presented in Fig. 3b. All the circles had the same radius, and their value is 5 mm. When one circle is placed in the left corner of the selected substrate, then the value of ${S}_{11}$ is − 10.75 dB at 9.97 GHz frequency, which is shown in step 1. In step 2, the circle is placed in the middle position on the substrate, then the gain of ${S}_{11}$ is − 22.04 dB at 9.95 GHz frequency. When two circles are placed in the left and middle positions of the substrate, then the magnitude of ${S}_{11}$ is − 27.28 dB at 9.57 GHz frequency, which is shown in step 3. At 9.92 GHz, the magnitude of ${S}_{11}$ is − 20.94 dB when two circles are put in the left and right corners of the substrate, shown in step 4. At 9.50 GHz, the magnitude of ${S}_{11}$ is − 23.13 dB when three circles are put in the left, middle and right locations of the substrate, shown in step5. In the final step three circles are placed in the left, middle, and right positions of the substrate, and the rectangular has covered them, then the magnitude of ${S}_{11}$ is − 23.87 dB at 9.46 GHz frequency.

**Figure 4:**
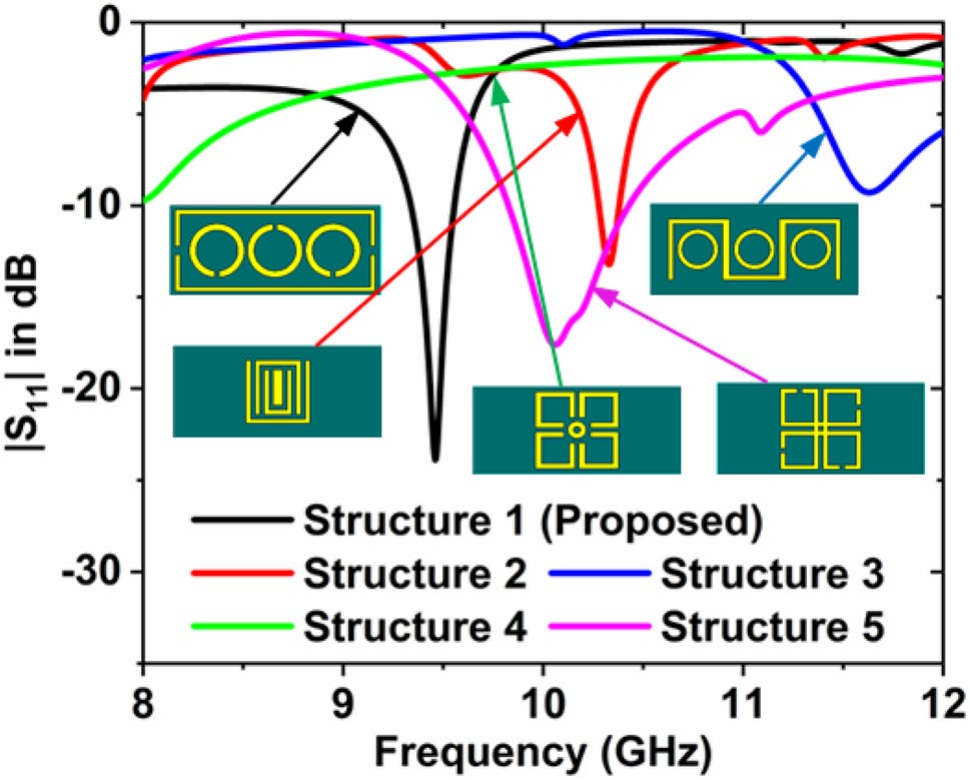
Frequency versus ${S}_{11}$ graph for the different resonator structures.

Figure 4 represents the frequency versus ${S}_{11}$ graph for the different resonator structures. There are five resonator structures used to design the proposed sensor. The magnitude of ${S}_{11}$ is − 23.87 dB at 9.46 GHz for structure 1, − 13.21 dB at 10.33 GHz for structure 2, − 9.29 dB at 11.63 GHz for structure 3, − 9.73 dB at 8 GHz for structure 4, and − 17.60 dB at 10.06 GHz for structure 5. The performance of structure 1 is better than others, so structure 1 is selected as a resonator pattern.

**Figure 5:**
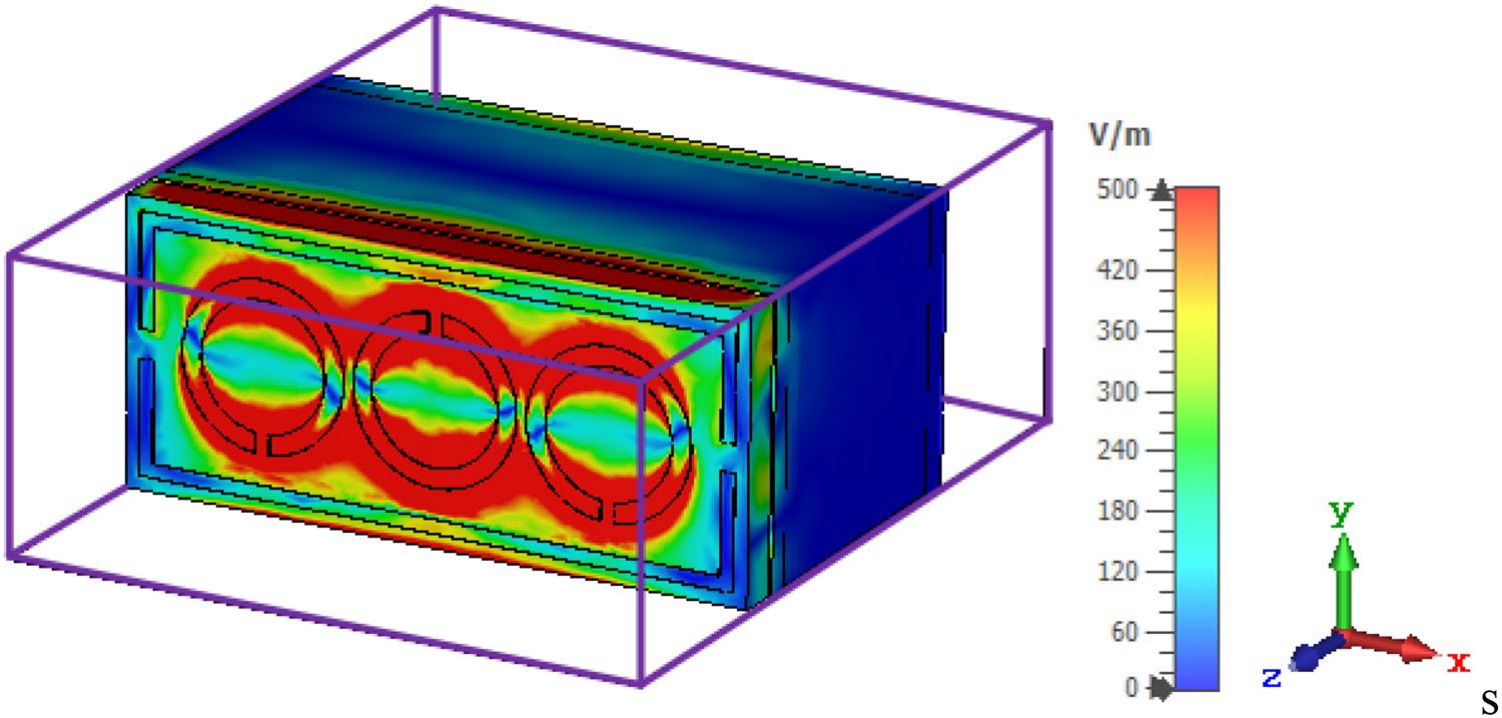
E-field distribution for the proposed MTM sensor.

At the resonance frequency of 9.46 GHz, the electric field distributions for the air are largely concentrated at the resonator surface shown in Fig. 5. The electromagnetic field is known to be able to travel across conductive cables. The field should be in transverse electromagnetic (TEM) mode, which means that the electric field is turned off. The distributions of electric field and surface current are also investigated to understand the designed sensor’s theory. Changes in the electric field and surface current distributions provide information about the device's energy contained and losses.

**Figure 6:**
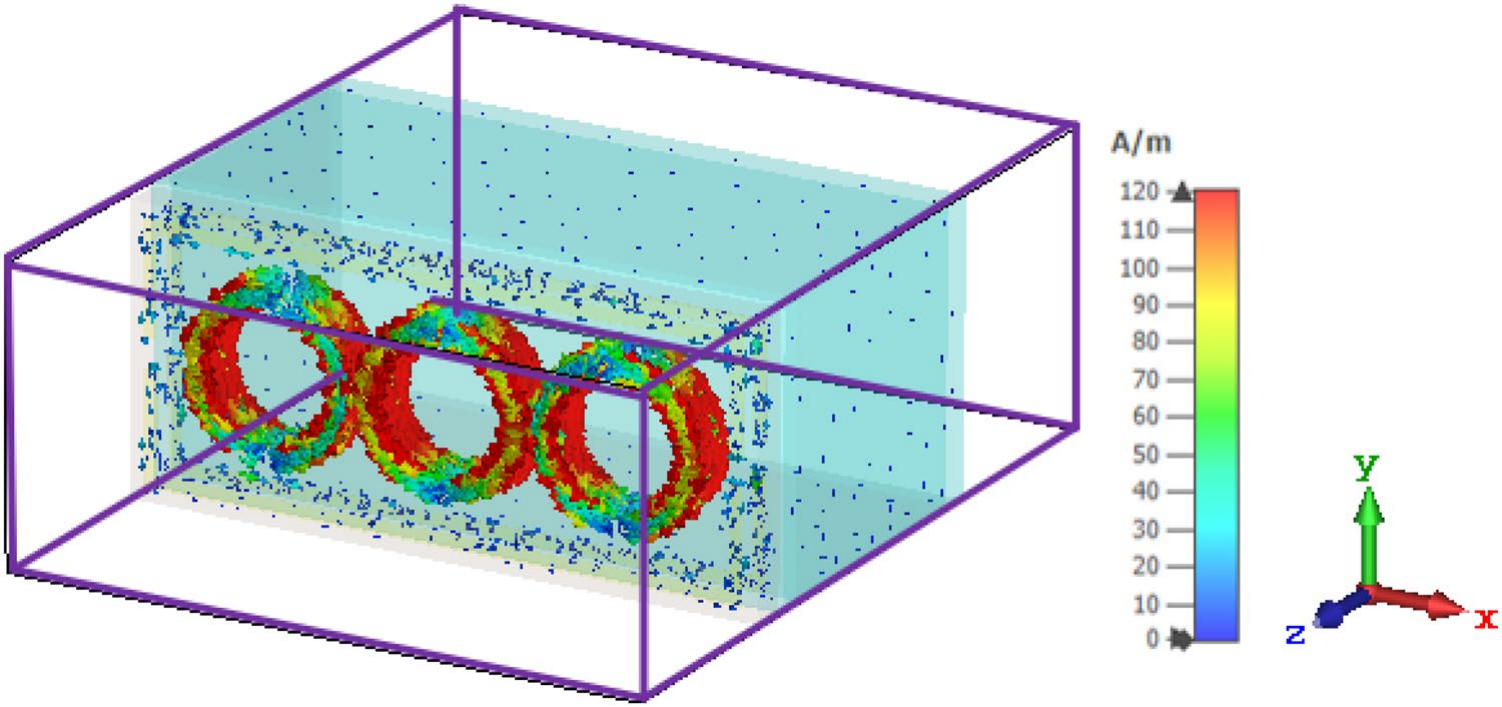
Distribution of surface current distribution for the sensor.

Figure 6 shows the circulation of surface current at the resonance frequency of 9.46 GHz. Surface current is more concentrated on the circle in the directions of clockwise and anti-clockwise. Furthermore, additional currents are dispersed on the left and right sides of the resonator, which regulates the electrical and magnetic responses. At the resonance frequency, the current concentration is equally in all circles. For the recommended structure, the existence of an electric dipole that creates a resonance event is demonstrated by the simulated surface current distribution.

**Figure 7:**
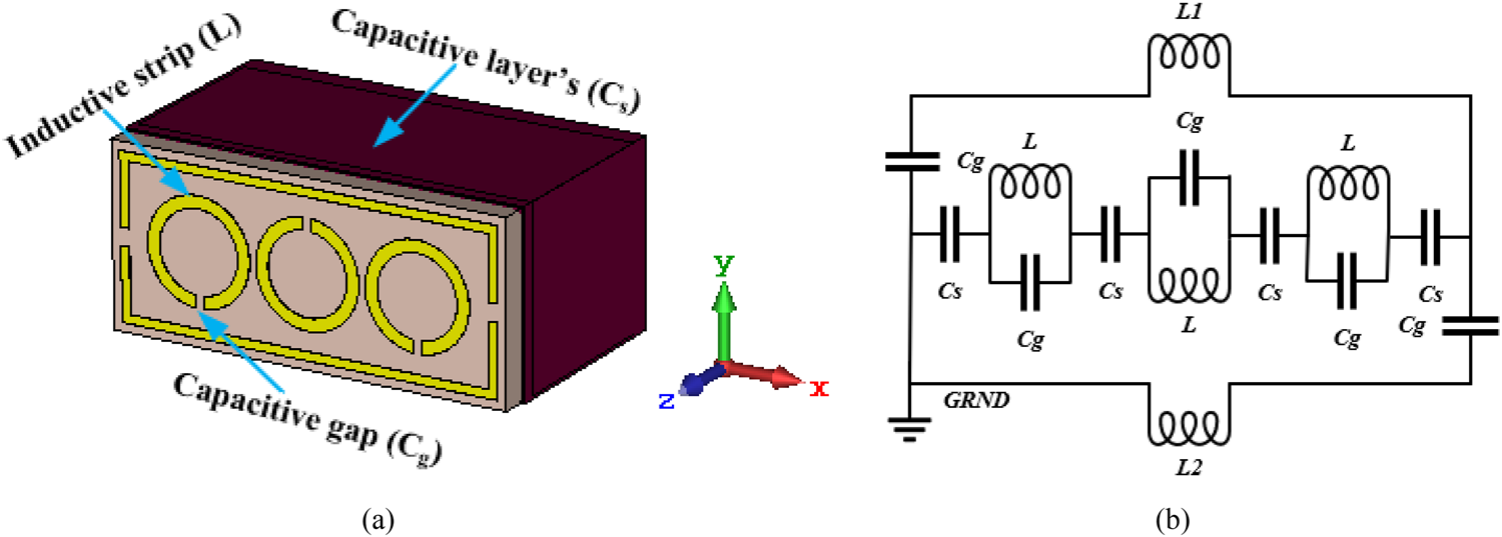
(**a**) Designed structure’s capacitive and inductive segments, (**b**) equivalent circuit of MTM sensor.

Figure 7a,b represents the capacitive and inductive segments and equivalent circuits of the designed MTM inspired sensor. The resonator can be represented by a total inductance (${L}_{t}$) and capacitance (${C}_{t}$). The resonator gaps denoted by the ${C}_{g}$, so the resonator can behave like a $LC$ model. Liquid samples with varying electrical characteristics can reduce the capacitance of the sensor layer $\left({C}_{s}\right)$ on the backside of the structure. The sensor layer’s capacitance $\left({C}_{s}\right)$ is shown in the following equations^48^

1 $${C}_{s}=\left(4A-g\right){C}_{pul}$$

here, $A$ is the average resonator dimension, $g$ is the gap of the split, and ${C}_{pul}$ is the capacitance per unit length. ${C}_{pul}$ can be computed as^51^

2 $$ C_{pul} = \frac{{\sqrt {\varepsilon_{r} } }}{{c_{0} Z_{0} }} $$

where, ${c}_{0}$ is the free space light speed and ${Z}_{0}$ is the line impedance. So, the structure's total capacitance can be expressed as,

3 $${C}_{t}={C}_{0}+{C}_{g}+{C}_{s}$$

here ${C}_{0}$ is the free space capacitance effect, ${C}_{g}$ is the capacitors for the gap, and ${C}_{s}$ is the effect of the sample’s capacitance put within the sensor layer. The value of ${C}_{s}$ can be varied for various samples, because of variations in the complex dielectric permittivity characteristics that can be stated by^23^

4 $$ \varepsilon_{sample} = \varepsilon_{sample}^{\prime } - j\varepsilon_{sample}^{\prime \prime } $$

where, $\varepsilon_{sample}^{\prime }$ is the real part and $\varepsilon_{sample}^{\prime \prime }$ is the imajinary part of permittivity.

The resonance frequency of the suggested structure can be determined using the following equation,

5 $$ f_{r} = \frac{1}{{2\pi \sqrt {L_{t} C_{t} } }} $$

here, ${L}_{t}$ is the structure’s overall inductance. It is clearly shown that the ${f}_{r}$ is inversely proportional to the ${L}_{t}$ and ${C}_{t}$ of the recommended resonator. So, the inductor and capacitor play an important role in defining the sensitivity of the sensor. It is mentioned that the basic operation of the sensor structure is correlated to the interaction of the resonator and the sensor layer.

## Parametric analysis of the metamaterial sensor

**Figure 8:**
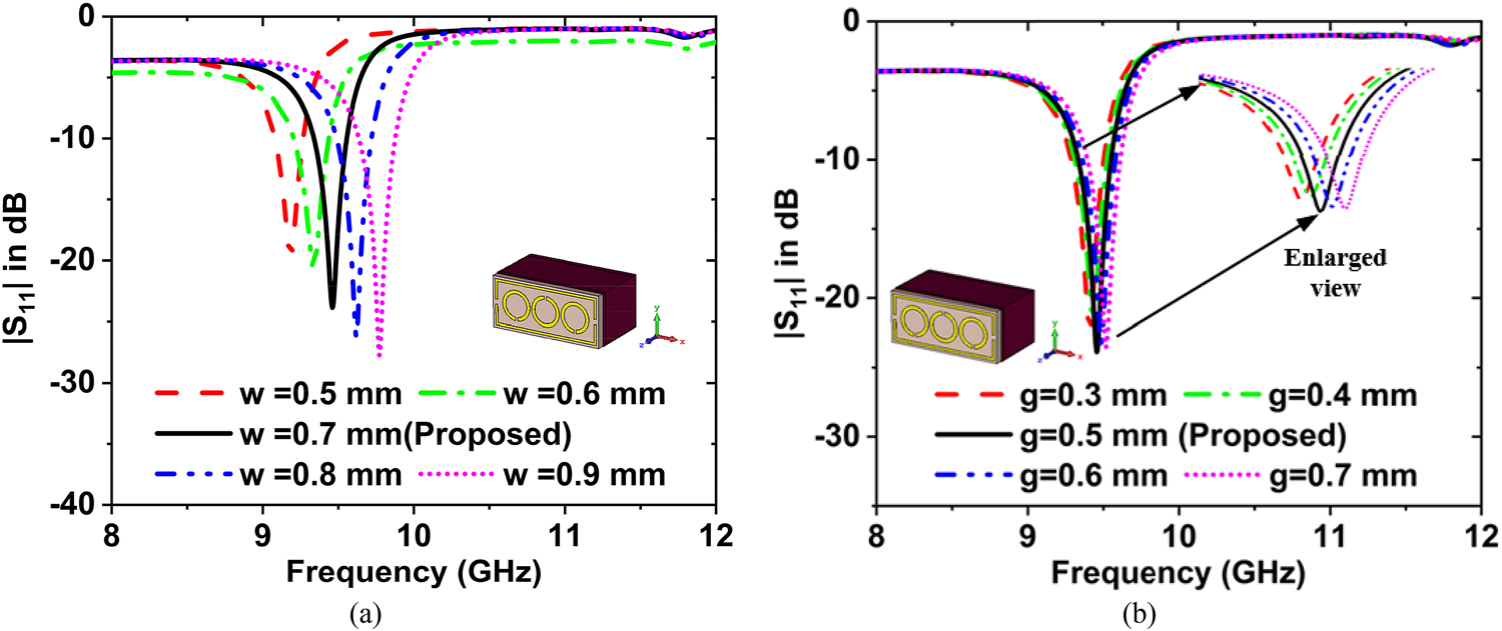
Effect on the resonance frequency for the change of (**a**) resonator width, and (**b**) resonator split gap.

The reflection coefficient (${S}_{11}$) and resonance frequency $\left({f}_{r}\right)$ are changing with the change of parameters i.e., resonator width (w), split gap (g), circle radius (r), sample holder thickness (SHT), substrate size (SS), and substrate material. In Fig. 8a the ${f}_{r}$ was more affected by the resonator width of the suggested sensor. In this MTM-inspired sensor, five different resonator widths have been used. If w = 0.5 mm, the ${S}_{11}$ value is − 19.76 dB at ${f}_{r}$ = 9.18 GHz. For w = 0.60, 0.70, 0.80. 0.90 mm, ${S}_{11}$ = − 20.73, − 23.87, − 26.21, and − 27.76 dB at ${f}_{r}$ = 9.34, 9.46, 9.62, and 9.77 GHz, respectively. The proposed MTM structure has a width of 0.6 mm.

The effect of the various gap of split on the ${f}_{r}$ as depicted in Fig. 8b. If the split gap g = 0.3 mm, then the ${S}_{11}$ value is − 22.34 dB at ${f}_{0}\hspace{0.17em}$= 9.41 GHz. For g = 0.4, 0.5, 0.6, and 0.7 mm, the values of ${S}_{11}$ are − 21.84, − 23.87, − 23.21, and − 23.51 dB at ${f}_{r}$ = 9.43, 9.46, 9.49, and 9.52 GHz, respectively. A split gap of 0.5 mm has been selected for the designed MTM sensor.

**Figure 9:**
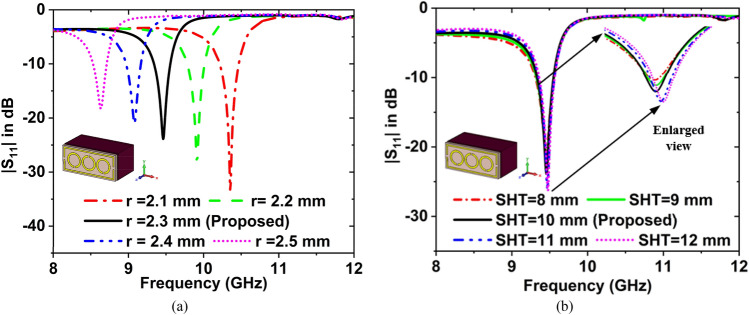
Effect on the resonance frequency for the change of (**a**) circle radius, and (**b**) sample holder thickness.

The effect of circle radius (r) on the $${f}_{r} $$ is shown in Fig. 9a. When taking the r = 2.1 mm, the value of $${S}_{11}$$ is − 33.69 dB at $${f}_{r}$$ = 10.35 GHz. For r = 2.2, 2.3, 2.4, and 2.5 the values of $${S}_{11}$$ are − 28.39, − 23.87, − 20.94, and − 18.26 dB, at $${f}_{r}$$ = 9.91, 9.46, 9.08, and 8.63 GHz, respectively. For the designed MTM sensor, a circle radius of 2.3 mm was used.

The ${f}_{r}$ is changes for the change of sample holder thickness (SHT), which is presented in Fig. 9b. For the SHT = 8, the magnitude of ${S}_{11}$ is − 21.08 dB at ${f}_{r}$ = 9.45 GHz. If the SHT = 9. 10, 11, 12 mm, then the magnitude of ${S}_{11}$ is − 22.35, − 23.87, − 26.18, and − 26.31 dB at ${f}_{r}$ = 9.46, 9.46, 9.47, and 9.48 GHz, respectively. The sample holder's size was set to 10 mm.

**Figure 10:**
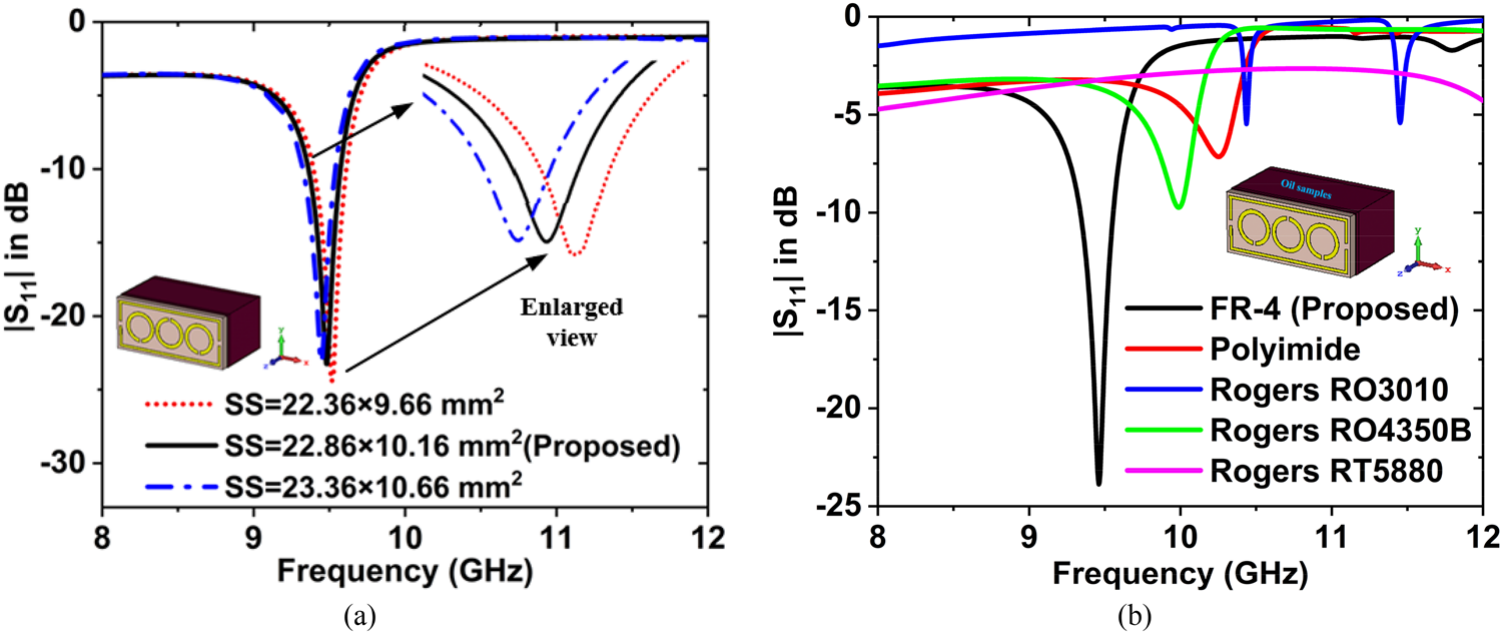
Effect on the resonance frequency for the change of (**a**) substrate size, and (**b**) substrate materials.

The substrate size’s (SS) effect on the $${f}_{r} $$ is shown in Fig. 10a. When taking the SS is 22.36 × 9.66 mm^2^, the value of $${S}_{11}$$ is − 24.40 at $${f}_{r}$$ = 9.5 2 GHz. For the 22.86 × 10.16 mm^2^ SS, the value of $${S}_{11}$$ is − 23.87 at $${f}_{r}$$ = 9.46 GHz. If the SS is 23.36 × 10.66 mm^2^, then the value of $${S}_{11}$$ is − 23.17 at $${f}_{r}\hspace{0.17em}$$= 9.45 GHz. Since the substrate size is directly proportional to the dielectric constant and $${f}_{r} $$ is inversely proportional to the dielectric constant hence when SS is increased $${f}_{r}$$ is decreased. Excitation is given through waveguide WR90 (90WCAS with SMA female output) attached on both sides to measure the reflection response ($${S}_{11}$$). 22.86 × 10.16 mm^2^ is the guided opening, so the SS of our designed MTM sensor is 22.86 × 10.16 mm^2^ has been chosen.

Figure 10b depicts the effect of the substrate materials on the resonance frequency. There are five substrate materials used to design the proposed sensor, these are FR-4, Polyimide, Rogers RO3010, Rogers RO4350B, and Rogers RT5880. The DK, LT, and thickness are 4.3, 0.025, and 1.5 for FR-4, 3.5, 0.0027, and 1.5 for Polyimide, 11.2, 0.0022, and 1.45 for Rogers RO3010, 3.66, 0.0037, and 1.524 for Rogers RO4350B, and 2.2, 0.0009, and 1.575 for Rogers RT5880. The magnitude of ${S}_{11}$ is − 23.87 dB at 9.46 GHz for FR-4, − 7.15 dB at 10.25 GHz for Polyimide, − 5.48 dB at 10.44 GHz, and − 5.42 at 11.45 GHz for Rogers RO3010, − 9.75 dB at 9.99 GHz for Rogers 4350B, and − 4.72 dB at 8 GHz for Rogers RT5880. The performance of the FR-4 substrate material is better than others, so FR-4 is selected as substrate material.

**Figure 11:**
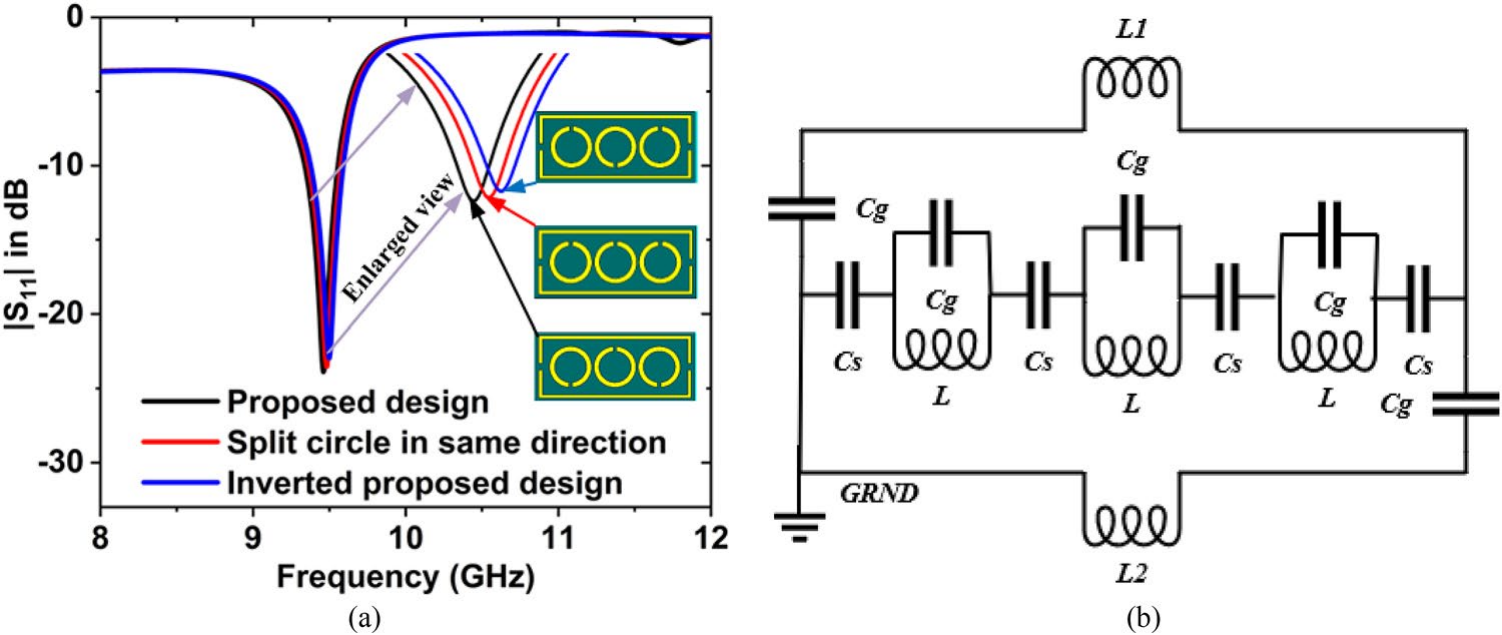
(**a**) ${S}_{11}$ graph for the different patterns of the circle split rings, (**b**) equivalent circuit for the split circle in the same direction.

Figure 11a illustrates the ${S}_{11}$ graph for the different patterns of the circle split rings. The magnitude of ${S}_{11}$ is − 23.87 dB at 9.46 GHz for the final design, − 7.15 dB at 10.25 GHz for the split circle in the same direction, and − 5.48 dB at 10.44 GHz, and − 5.42 at 11.45 GHz for the inverted split circle rings of the proposed design. The equivalent circuit is drawn when the circle split ring faces in the same direction which is presented in Fig. 11b. From this circuit, it is seen that the capacitive effect is reduced because the circle split capacitors and inductors are on the same side and lumped effect. Hence the resonance frequency is slightly increased. Aso, when the proposed circle split rings pattern, is inverted the mutual coupling capacitor effect is decreased and the resonance frequency is increased which is shown in Fig. 11a.

## Results and discussions

**Figure 12:**
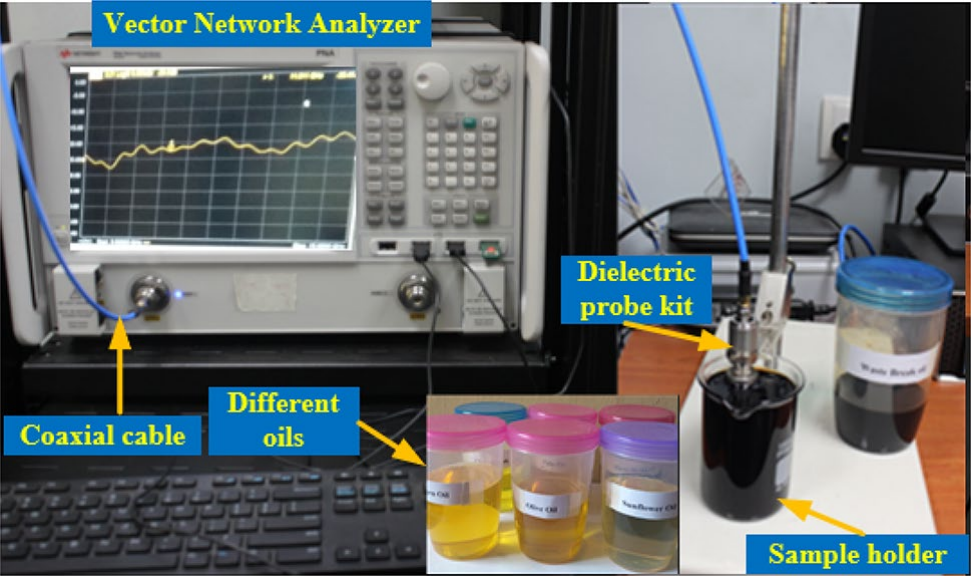
Measurement setup for the DK and LT of different samples using the N1500A dielectric probe kit.

Figure 12 represents the dielectric constant (DK) and loss tangent (LT) measurement procedure for the different samples. The permittivity values and LT of the oils, fluids, and chemicals were determined using a calibrated Agilent network analyzer and the N1500A dielectric probe kit.

**Figure 13:**
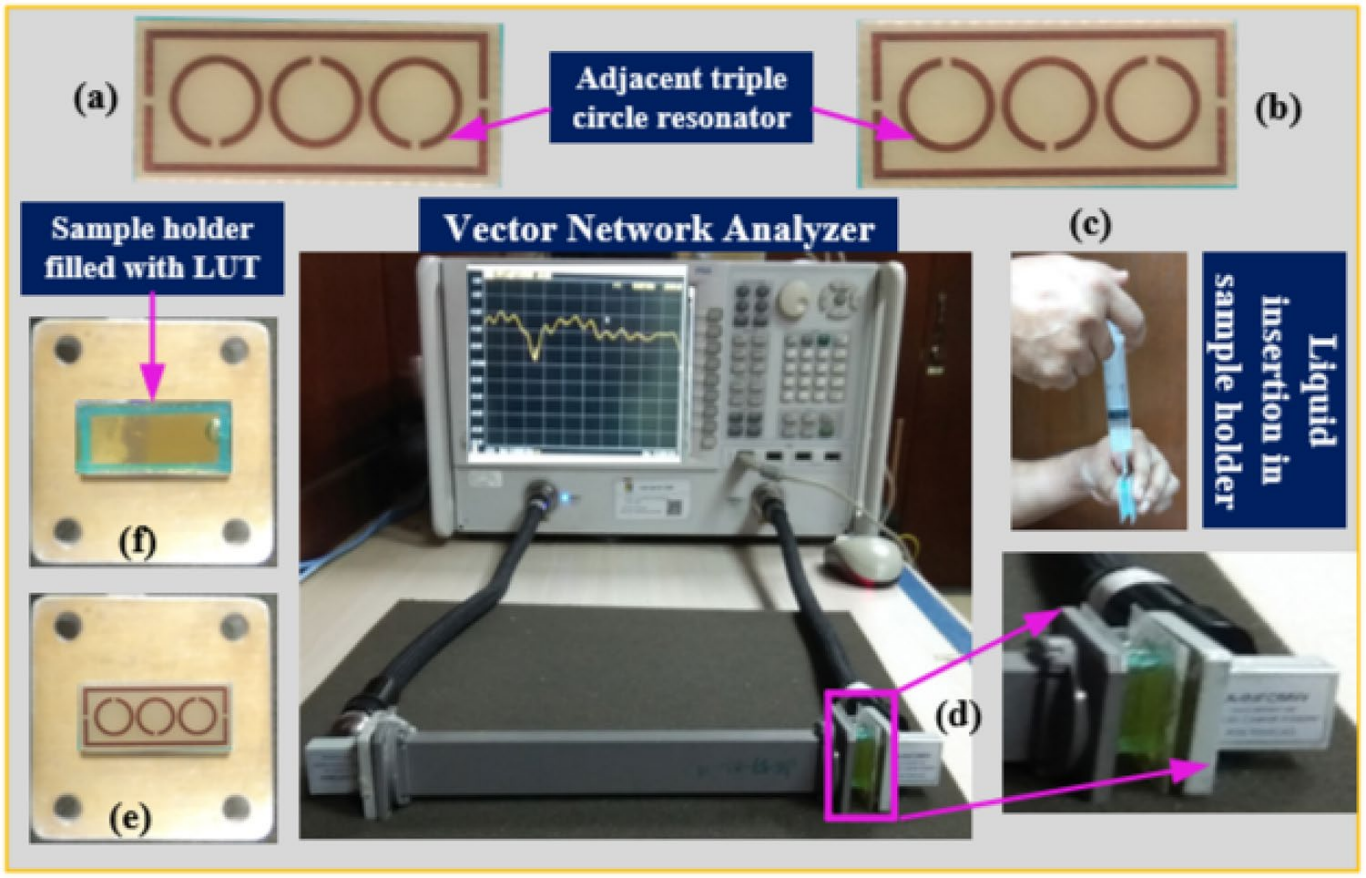
(**a**) Front view of fabricated MTM based sensor (**b**) back view (**c**) process of liquid insertion (**d**) experimental setup using waveguide (**e**) sensor attached with X-band waveguide and (**f**) sample holder attached with an extended guided wave.

**Figure 14:**
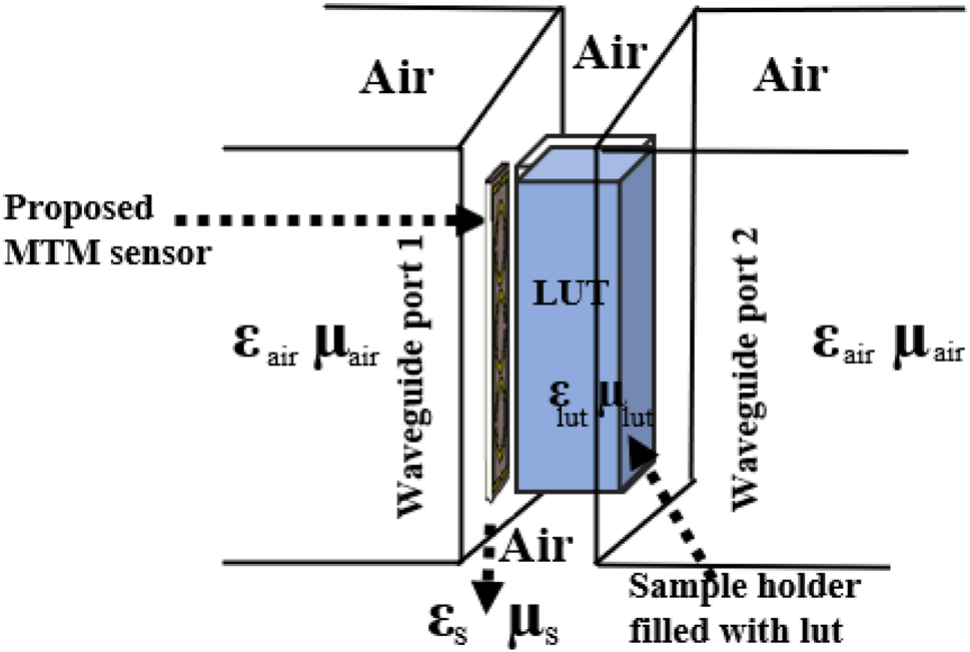
Schematic view of the microwave sensing measurement setup.

Figure 13a–f depicts the suggested MTM sensor's entire measurement procedure. After the design process, the proposed sensor was developed using LPKF Laser and Electronics AG, Computerized Numerical Control (CNC) model Promat E33, and printed circuit board (PCB) machines, as shown in Fig. 13a,b. The container is filled with different liquid under test (LUT) samples and attached with the suggested structure. Each sample has been filled with a different holder to avoid contaminations between the samples. The Agilent N4694-60001 calibration kit was used to calibrate the PNA series vector network analyzer (VNA type N5227A). The coaxial cables link the VNA to the two waveguides. One waveguide is a transmitter, while the other is a receiver. With the sample holder, adaptor, waveguide, and sensor structure present, the ${S}_{11}$ was measured. First, the sample was injected into the sample holder, and then, in the target frequency range of 8–12 GHz, the ${S}_{11}$ was determined. Figure 13c reveals the liquid incorporation process in the sample holder, Fig. 13d shows the ${S}_{11}$ measurement system using waveguide, Fig. 13e,f represents the MTM sensor and sample holder connected with the waveguide. Figure 14 shows the schematic view of the microwave sensing measurement setup. Some fluctuations of oscillating shape have been seen on the VNA during the measurement. These fluctuations occurred due to the waveguides coupling effect and the MTM sensor fabrication tolerance during the prototype production of the FR-4 substrate layer. The dielectric constant of the substrate layer and testing environment have also influenced these fluctuations.

**Figure 15:**
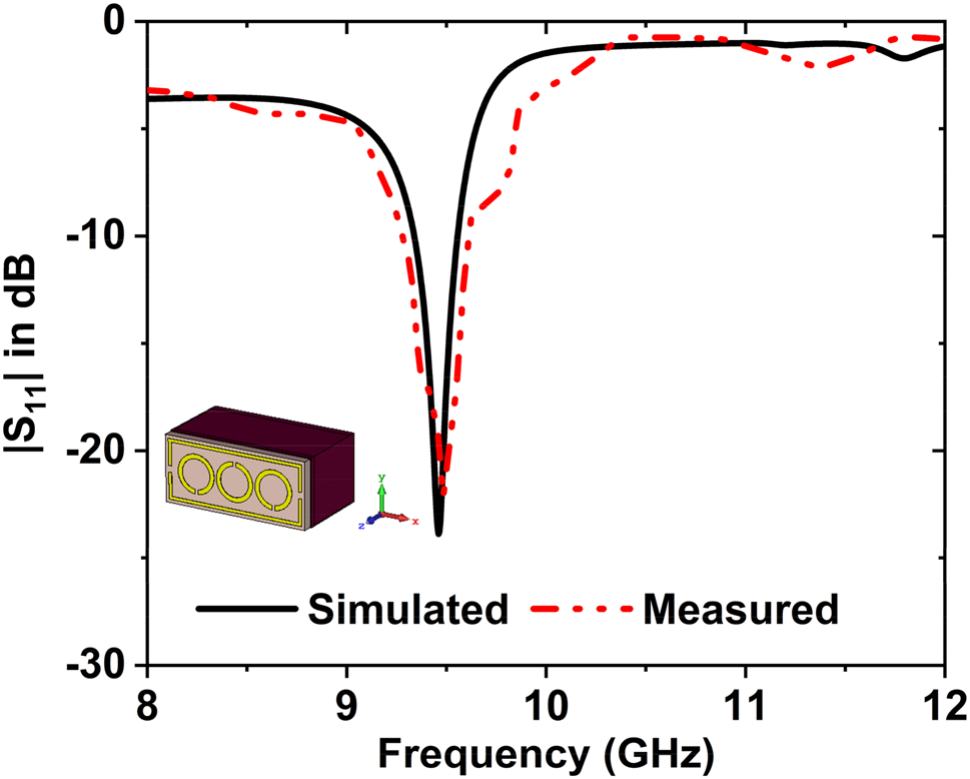
${S}_{11}$ results when the sample holder is filled with air.

The measured and simulated reflection coefficient graph is depicted in Fig. 15, where the sample holder was filled with air. The value of ${S}_{11}$ is − 23.87 dB at 9.46 GHz in the simulation, and − 22.17 dB at 9.48 GHz in the measurement.

### Study on the detection of olive oil and corn oil

**Figure 16:**
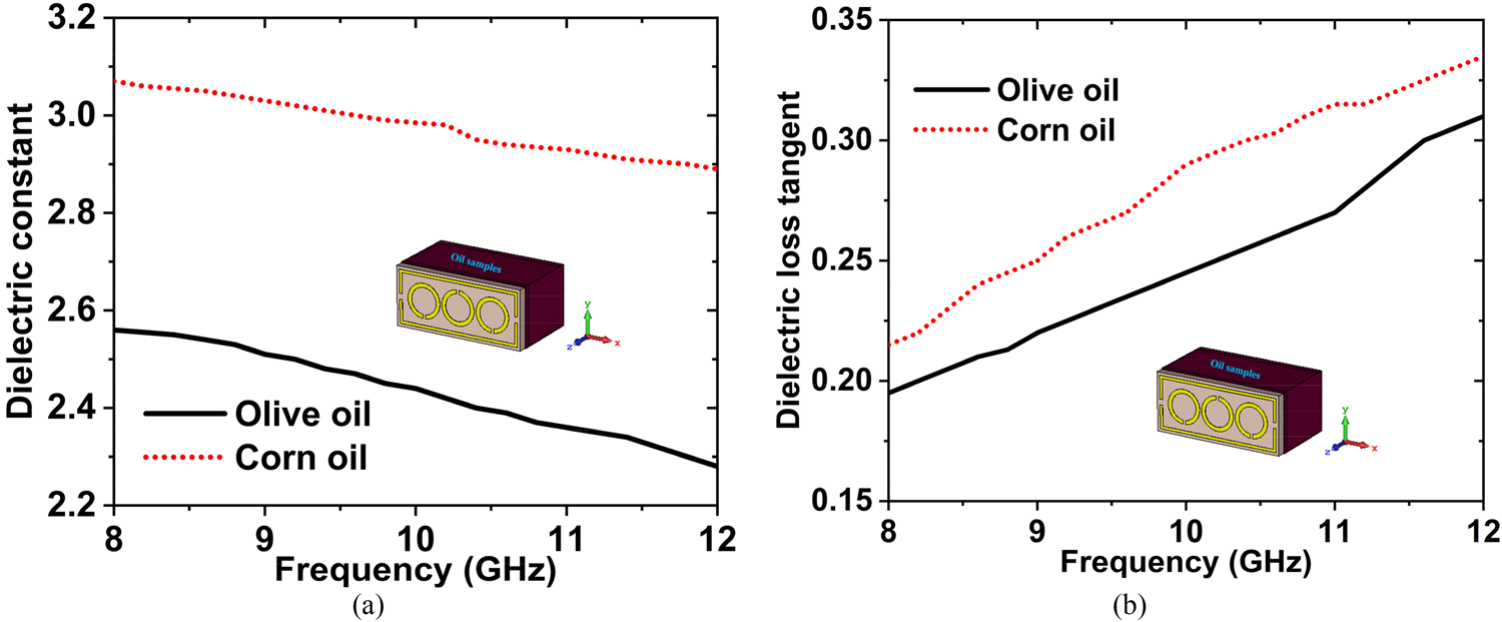
Measured (**a**) DK and (**b**) LT for the corn oil and olive oil.

Olive oil is a cooking oil obtained from olives, which are the fruit of the olive tree. It is most commonly used for high blood pressure, high cholesterol, and heart disease. This oil is also used in cooking and salad dressings. Corn oil is produced from the germ of the corn plant (maize). It is a refined vegetable oil that's commonly used in cooking, particularly deep frying. It is less expensive than the majority of other vegetable oils. Firstly, we have collected the olive and corn oils from the nearest super shop, then measured the DK and LT in the microwave lab using the dielectric measurement kit. After measuring the DK and LT of these oils, we have made the graphs. The graphs have been plotted using the obtained measured data, which is shown in Fig. 16. From the Figure, we can see that the value of DK is decreasing with increasing frequency, whereas the value of LT is increasing with increasing frequency. These phenomena show that, the DK and ${f}_{r}$ are inversely proportional, where the LT and ${f}_{r}$ are directly proportional. The measured DK is 2.56 and LT is 0.197 for olive oil at the 8–12 GHz range of frequency, where the DK is 3.07 and LT is 0.208 for the corn oil at that range of frequency.

**Figure 17:**
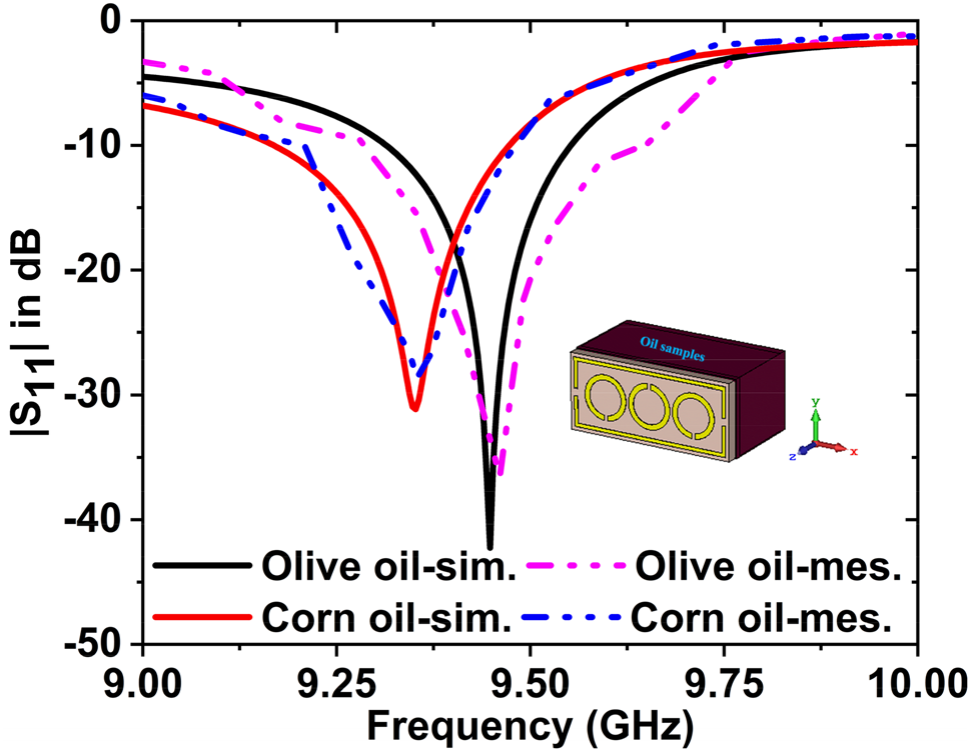
${S}_{11}$ results for the corn oil and olive oil.

The ${S}_{11}$ data for olive and corn oils have been plotted in Fig. 17. The simulated and experimental resonance frequencies are 9.45 GHz and 9.46 GHz for the olive oil with − 42.25 dB and − 36.59 dB magnitude, respectively. For corn oil, the magnitude values are − 31.51 dB at 9.35 GHz & − 28.67 dB at 9.35 GHz for the simulated and measured, respectively. The ${f}_{r}$ has been shifted to 100 MHz with − 10.74 dB magnitude change (simulated) and 110 MHz shifted with − 7.92 dB magnitude change (measured) for these two oils. By changing the resonance frequency, we can detect the presence of various oils. These findings show that despite their dielectric behaviour being very similar, the proposed MTM sensor accurately detects a variety of oils. The results of the measured and simulated are very similar. However, some of the fabrication and calibration errors produced minor discrepancies between them.

**Figure 18:**
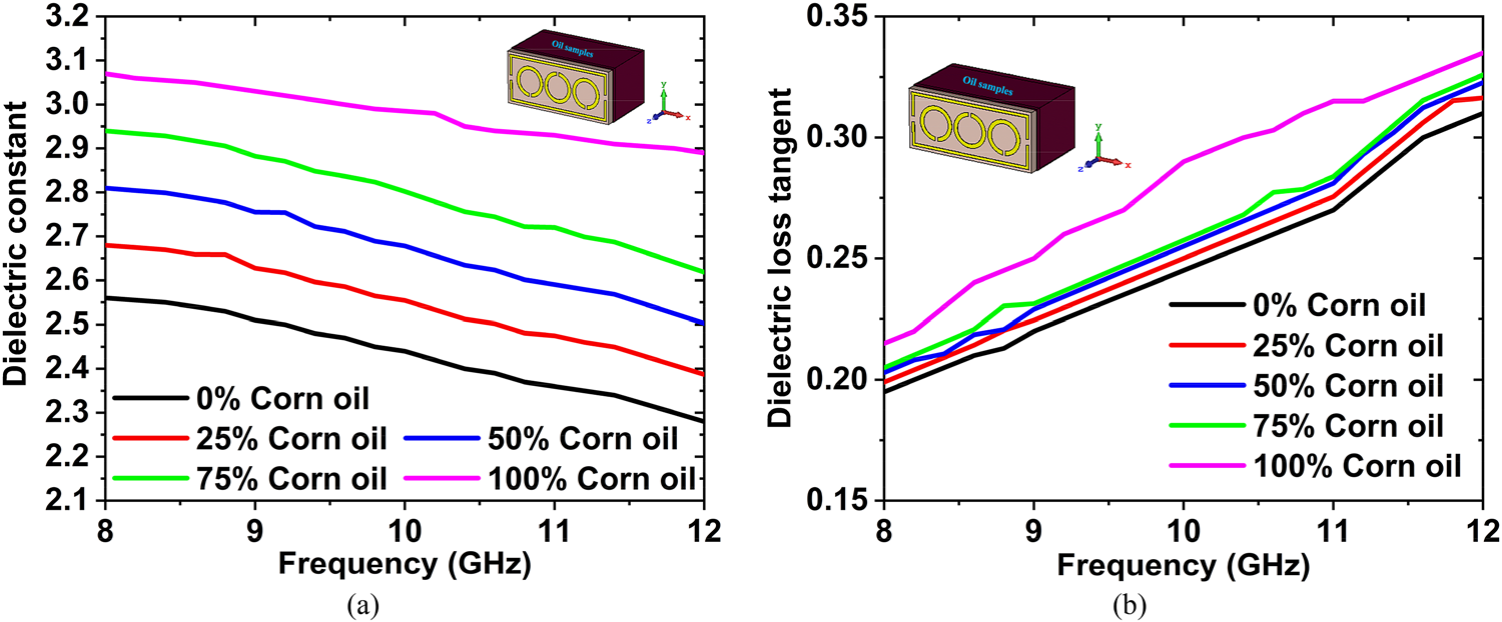
Measured (**a**) Dielectric constant, and (**b**) Loss tangent curves for the different concentration (0%, 25%, 50%, 75%, and 100%) of corn oil in olive oil.

Firstly, different concentration (0%, 25%, 50%, 75%, and 100%) of corn oil has been mixed with olive oil, then the dielectric constant and loss tangent of these mixtures are measured by using the dielectric probe kit. The DK and LT graphs have been plotted by using the obtained measured data. Figure 18a,b represents the DK and LT graphs for the different concentrations of corn oil in olive oil. The measured DK are 2.56, 2.68, 2.81, 2.94, 3.07 and LT are 0.195, 0.199, 0.203, 0.205, and 0.21 at 8 GHz.

**Figure 19:**
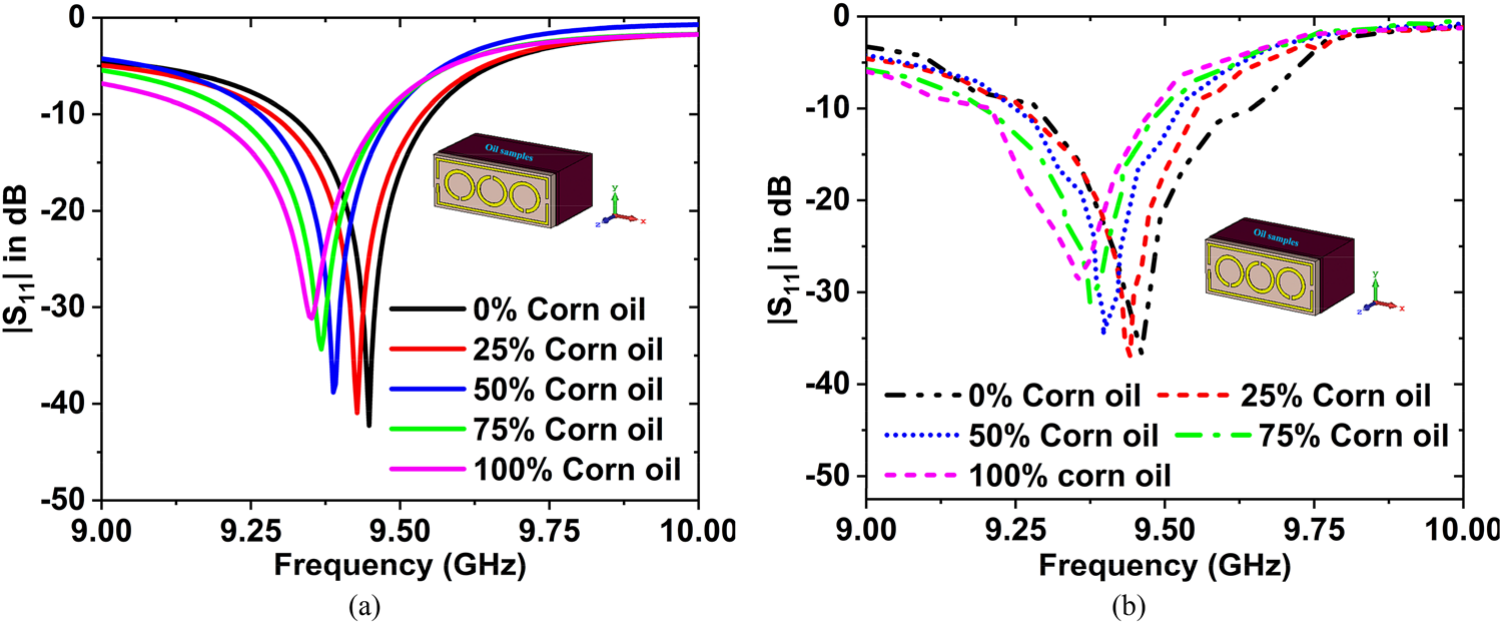
(**a**) Simulated, and (**b**) Measured ${S}_{11}$ curves for the different concentration (0%, 25%, 50%, 75%, and 100%) of corn oil in olive oil.

The impact of different concentrations of two samples has been also studied. The simulated resonance frequencies are 9.45, 9.43, 9.39, 9.37, and 9.35 GHz with magnitudes of ${S}_{11}$ are − 42.25, − 40.92, − 38.79, − 34.33, and − 31.13 dB for the 0%, 25%, 50%, 75%, and 100% concentration of corn oil in olive oil. The measured resonance frequencies are 9.46, 9.44, 9.40, 9.37, and 9.35 GHz with magnitudes of ${S}_{11}$ are − 36.59, − 36.91, 34.63, − 31.06, and − 28.67 dB for the 0%, 25%, 50%, 75%, and 100% concentration of corn oil in olive oil. Figure 19a,b depicts the simulated and measured results for the 0%, 25%, 50%, 75%, and 100% concentration of corn oil in olive oil.

### Study on the detection of sunflower oil and palm oil

**Figure 20:**
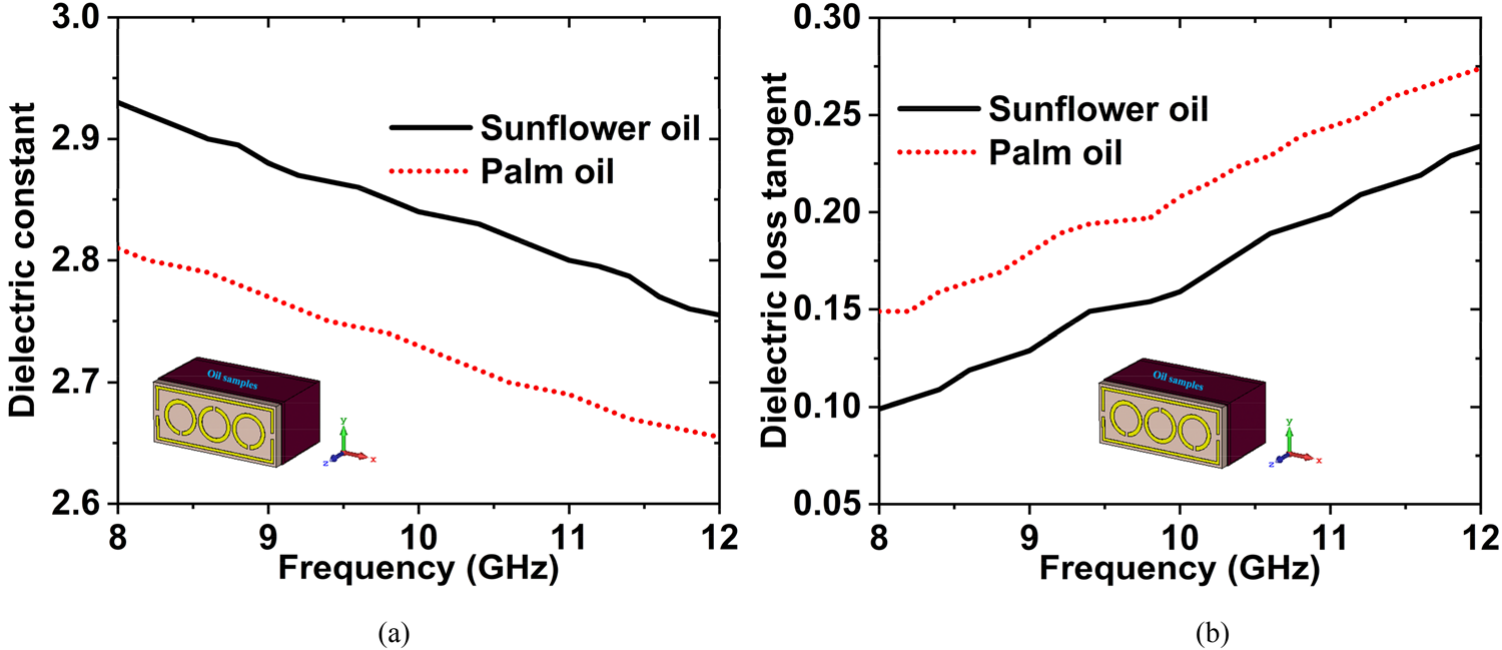
Experimental data of (**a**) DK (**b**) LT for the sunflower & palm oils.

Sunflower oil is a non-volatile oil obtained from the seeds of sunflowers. It's widely utilized in the culinary industry as a frying oil and as a cosmetic emollient. It is used for constipation and lowering "bad" LDL cholesterol. Palm oil is an edible vegetable oil derived from palm tree fruit. Vitamin A deficiency, cancer, brain disorders, and aging are all prevented by this supplement. Firstly, we have collected the sunflower oil and palm oil from the nearest super shop, then measured the dielectric constant (DK) and loss tangent (LT) in the microwave lab using the dielectric measurement kit. After measuring the DK and LT of these two oils, we have made the graphs. The graphs have been plotted using the obtained measured data, which is shown in Fig. 20. The DK and LT values are 2.93 and 0.099 for sunflower oil, whereas 2.81 and 0.149 for palm oil at 8 GHz frequency.

**Figure 21:**
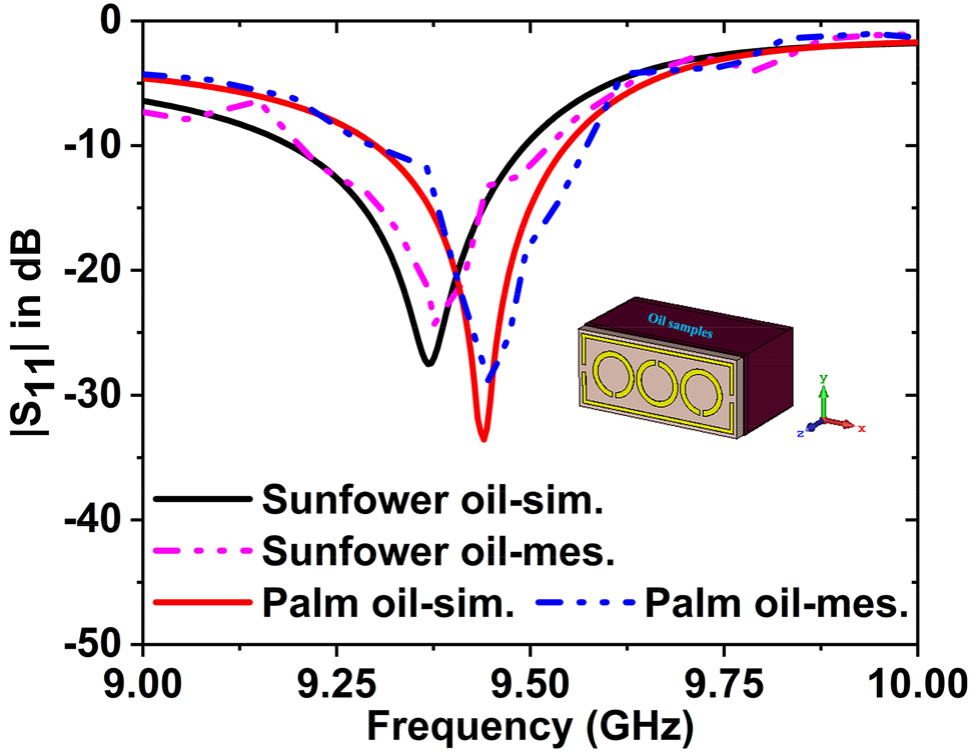
${S}_{11}$ results for the palm oil and sunflower oil.

The simulated and experimental ${S}_{11}$ data for palm oil & sunflower oil have been plotted in Fig. 21. The simulated and experimental resonance frequencies are 9.36 GHz and 9.37 GHz for the sunflower oil with − 27.54 dB and − 24.30 dB magnitude, respectively. For palm oil, the magnitude values are − 33.53 dB at 9.44 GHz and − 28.95 dB at 9.45 GHz for the simulated and measured, respectively. It is noticeable that the ${f}_{r}$ has been shifted to 80 MHz with − 5.99 dB magnitude change (simulated) and 80 MHz shifted with − 4.65 dB magnitude change (measured) for these two oils. By changing the resonance frequency, we can detect the presence of various oils. The results of the measured and simulated are very similar. However, some of the fabrication and calibration errors produced minor discrepancies between them.

### Study on the detection of clean and waste brake fluids

**Figure 22:**
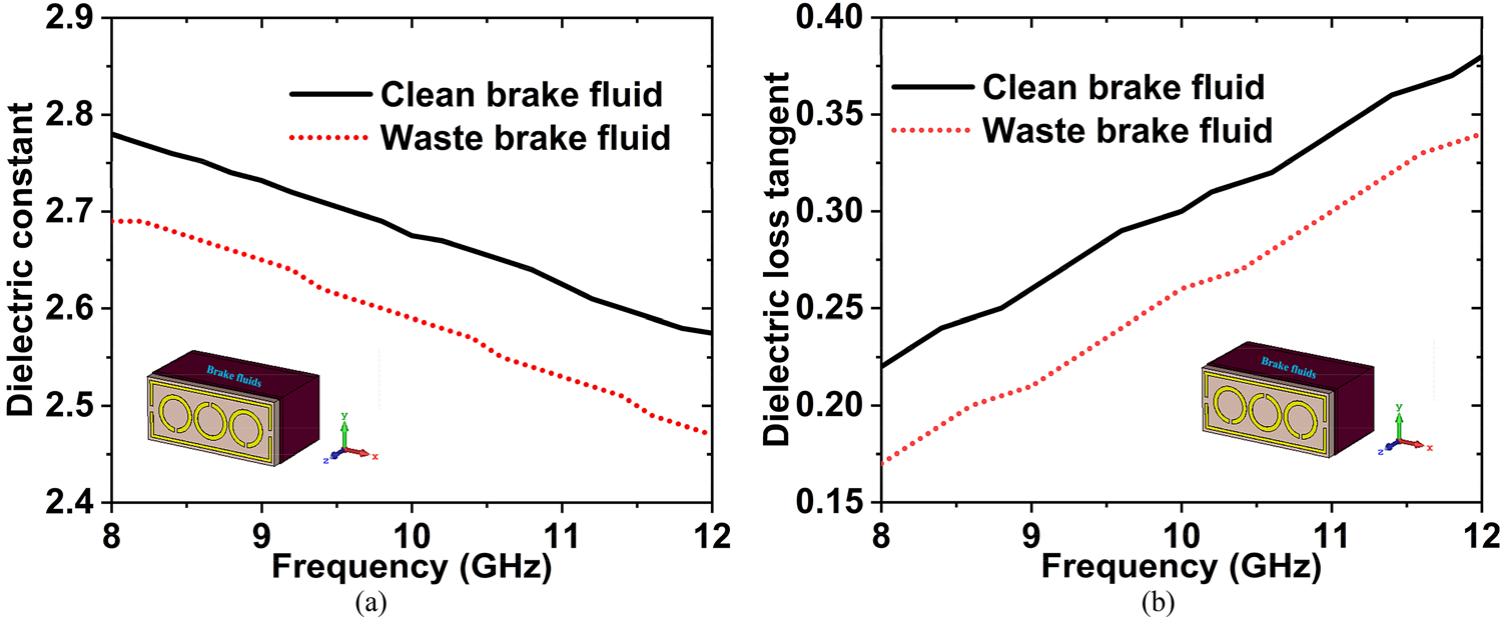
Experimental data of (**a**) DK and (**b**) LT for the waste & clean brake fluids.

Brake fluid is crucial to making sure that brakes run smoothly while driving the vehicle. It needs to be new and full to allow it to brake properly and keep safe. Sometimes, the brake does not work properly with the waste brake fluids, so, need to know the condition of the brake fluids. Firstly, we have collected the clean and waste brake fluid from the nearest motor repairing workshop, then measured the dielectric constant (DK) and loss tangent (LT) in the microwave lab using the dielectric measurement kit. After measuring the DK and LT of these two fluids, we have made the graphs. The graphs have been plotted using the obtained measured data, which is shown in Fig. 22. The DK and LT values are 2.78 and 0.22 for clean brake fluid, where 2.69 and 0.17 for waste brake fluid at 8 GHz frequency.

**Figure 23:**
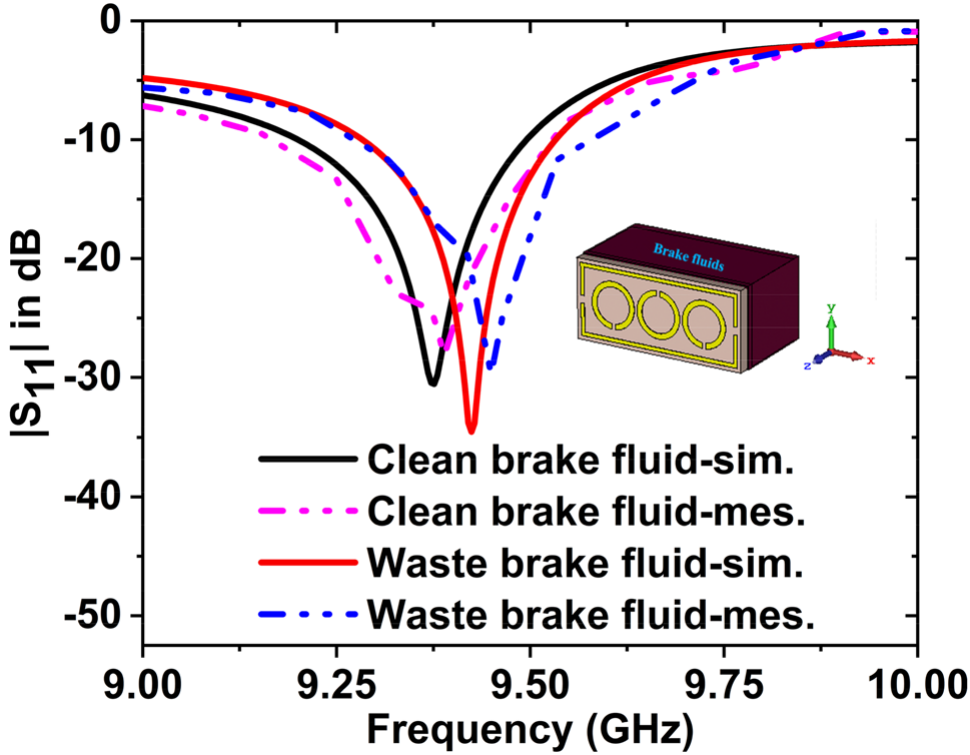
${S}_{11}$ result for the waste and clean brake fluids.

The simulated and measured ${S}_{11}$ data for clean and waste brake fluids have been plotted in Fig. 23. Since the simulated resonance frequency of the clean and waste brake fluids are 9.37 GHz with − 30.55 dB and 9.44 GHz with − 34.52 dB, and measured values are 9.38 GHz, with − 28.13 dB, and 9.44 GHz with − 29.74 dB. Hence the resonance frequency shifted to 70 MHz (simulated) and 60 MHz (measured) with magnitude changes of − 3.97 dB and − 1.61 dB, respectively. By changing the resonance frequency, we can detect the presence of various oils. The measured and simulated outcomes are in a nice contract with each other. However, a few of the fabrication and calibration errors produced minor discrepancies between them.

### Study on the detection of benzene and carbon-tetrachloride

**Figure 24:**
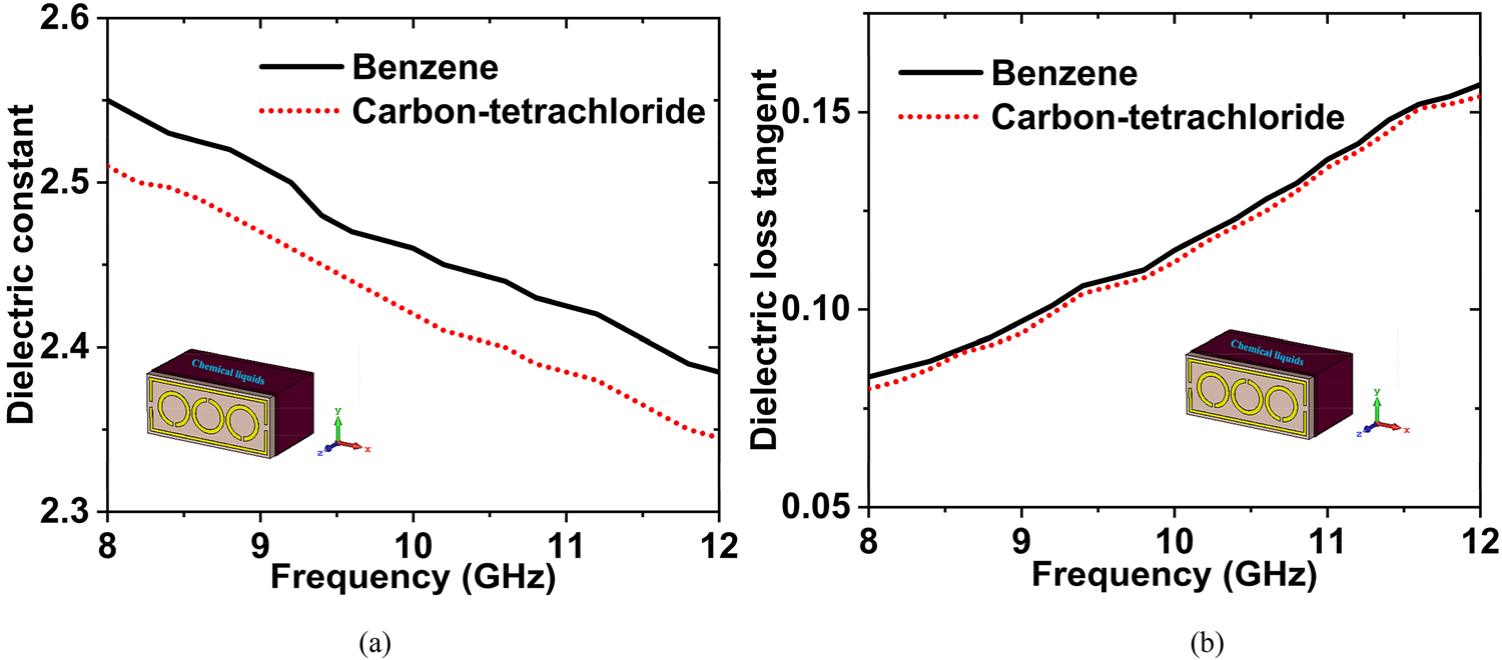
Experimental data of (**a**) DK (**b**) LT for the carbon-tetrachloride & benzene.

Benzene is the most fundamental organic aromatic hydrocarbon and the parent compound of many aromatic compounds. It's a colorless liquid with a feature primarily utilized to make polystyrene. It's used to make plastics, resins, synthetic fibers, rubber lubricants, colors, detergents, medications, and pesticides. Carbon-tetrachloride is a synthetic compound that does not exist in nature. It's a clear liquid with a sweet smell that's detectable at low concentrations. It's utilized as a rubber solvent, a cleaning agent in the dry-cleaning business, and a solvent in the chemical and medicinal industries. Firstly, we have collected the benzene ad carbon-tetrachloride from the nearest chemical shop, then measured the DK and LT in the microwave lab using the dielectric measurement kit. After measuring the DK and LT of these two chemicals, we have made the graphs. The graphs have been plotted using the obtained measured data, which is shown in Fig. 24. The DK and LT values are 2.55 and 0.083 for benzene, whereas 2.51 and 0.079 for carbon-tetrachloride at 8 GHz frequency.

**Figure 25:**
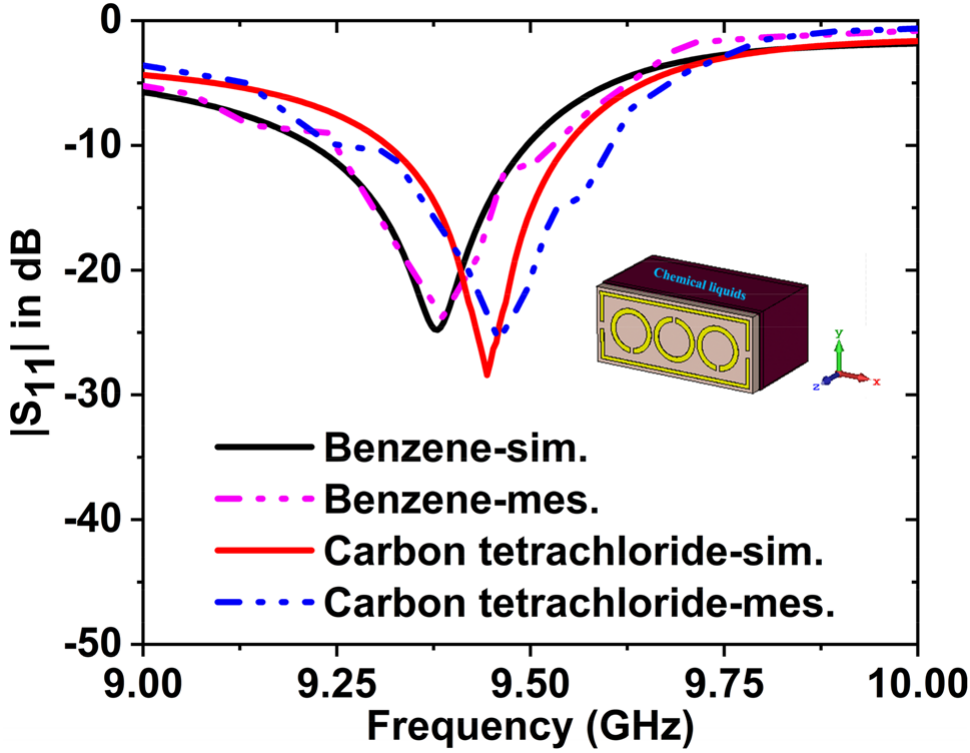
${S}_{11}$ results for benzene, and carbon tetrachloride.

The simulated and measured ${S}_{11}$ data for benzene and carbon tetrachloride have been plotted in Fig. 25. The simulated and experimental resonance frequencies are 9.38 GHz and 9.39 GHz for the benzene with − 24.79 dB and − 23.77 dB magnitude, respectively. For carbon tetrachloride, the magnitude values are − 28.41 dB at 9.46 GHz, − 25.62 dB at 9.48 GHz. The ${f}_{r}$ has been changed to 80 MHz with − 3.62 dB magnitude change (simulated) and 90 MHz shifted with − 1.85 dB magnitude change (measured) for these two oils. By changing the resonance frequency, we can detect the presence of various oils. The experimental and simulated findings are in a great deal with each other. However, some of the fabrication and calibration errors produced minor discrepancies between them.

When the concentration & DK of the samples are changed, the reflection response $({S}_{11})$ varies. Minor discrepancies exist between simulated and measured results of ${S}_{11}$ due to the mutual coupling effect of the waveguide port, fabrication tolerance, and calibration inaccuracy. In addition, the mutual resonance effect of two waveguide ports' transmitting and receiving ends will always affect the readings and produce minor variations in both readings.

The accuracy of the sensor is the maximum difference that will exist between the actual value and the indicated value at the output of the sensor. Firstly, the dielectric permittivity of the liquid samples is measured by using the dielectric probe kit, then the reflection coefficients of the sensor are measured for the liquid samples. Then the linear curve fitting equation is formed by using the obtained resonance frequency with corresponding measured dielectric permittivity. The linear curve fitting equation is

6 $$ \varepsilon_{sample} = - 4.1746f_{r} + 41.955 $$

here ${f}_{r}$ is the resonance frequency.

Now the measured dielectric constant of the olive oil is 2.46 at 9.46 GHz and the dielectric permittivity is obtained from the sensor is 2.48 at 9.46 GHz (using Eq. 6), again the dielectric constant of the clean brake fluid is 2.77 at 9.38 GHz and the dielectric permittivity is obtained from the sensor is 2.79 at 9.38 GHz which is very close to the measured value. So, it can be said that the accuracy of the sensor is superior.

**Figure 26:**
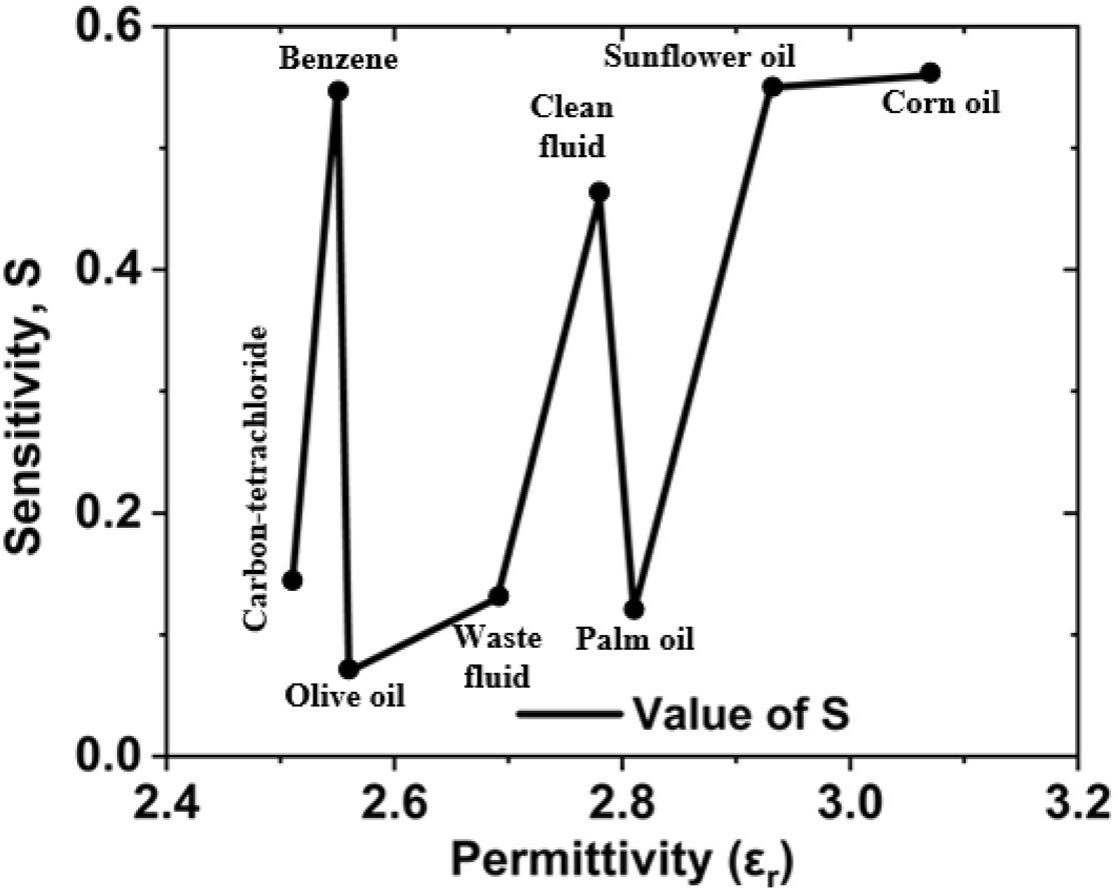
Sensitivity vs permittivity curve for the suggested MTM sensor.

The extracted sensitivities $S\left(\%\right)$ is defined as follows^34,52^:

$$S\left(\%\right)=\frac{f-{f}_{0}}{{f}_{0}({\varepsilon }_{r}-1)}\times 100$$

where, $f$ is the initial frequency (when sample holder is empty), ${f}_{0}$ is the frequency when the sample holder is filled one after the material change and ${\varepsilon }_{r}$ is the permittivity value of the materials. Figure 26 represents the sensitivity vs permittivity curve.

The quality-factor is a most important aspect of a metamaterial sensor to investigate the dielectric property. A common concern is that most MTM sensors suffer from low quality-factor and high measurement errors, and this, in turn, limits their usage in many applications. The quality-factor for the proposed sensor is, $Q={f}_{r}/\Delta f$^53,54^, here ${f}_{r}$ is the resonance frequency and $\Delta f$ is the + 3 dB bandwidth and it is dimensionless since the unit of both parameters are same. The difference between the two values of the independent variable at which the dependent variable equals 70.7% with respect to the minimal reflection value is known as the + 3 dB bandwidth. Again $\Delta f={f}_{h}-{f}_{l}$, ${f}_{h}$ is the higher cutoff frequency, and ${f}_{l}$ is the lower cutoff frequency at 70.7% of its minimal reflection value. The Q-factor has been extracted by using this formula and the obtained value is 135. The maximum quality-factor was discovered via the olive oil simulation and experimental analysis. The quality-factor 135 has been found in the proposed metamaterial sensor.

As a quantitative description of the sensing performance characteristic, we calculated the figure of merit (FOM), which is defined as $FoM=S\times Q$, where $S$ is the sensitivity and $Q$ is the quality factor^55^.

The sensitivity of the proposed sensor is $S=0.56$ and the quality factor $Q=135$, hence the $FoM=0.56\times 135=75.6\approx 76$

The frequency selectivity mechanism based on the concentration and purity of the liquid samples is used in the proposed sensor. The property of the sensor enables it to be “tuned” to respond better to certain frequencies than to others. The degree of selectivity of such filtering is sometimes specified as Q, which is the center or best frequency of a filter divided by its bandwidth. The proposed sensor identifies the concentration and purity of the liquid samples since the resonance frequency is shifted according to dielectric permittivity variation and the dielectric permittivity depends on the concentration and purity of the recommended liquid samples.

A comparison between measured DK and existing literature is indicated in Table 1.

**Table 1:** Comparison of dielectric constant (DK) values between measured and existing works.

Material sensing	DK (measured) at 8 GHz	Dielectric constant (reported literature)	
		DK	References
Olive oil	2.56	2.55 at 8 GHz	^48^
Corn oil	3.07	3.08 at 8 GHz	^48^
Sunflower oil	2.93	3.08 at 1 MHz	^56^
Palm oil	2.81	3.03 at 1 MHz	^56^
Clean brake fluid	2.78	2.36 at 8 GHz	^47^
Waste brake fluid	2.69	2.25 at 6 GHz	^47^
Benzene	2.55	2.57 at 8 GHz	^48^
Carbon-tetrachloride	2.51	2.52 at 8 GHz	^48^

Table 2 shows the comparison among the proposed MTM inspired sensor and other sensors mentioned in the literature regarding the sensing materials, dielectric constant, the shift of ${f}_{r}$, change of magnitude, Q-factor,and FOM. From references^20,23,27,28,36–38,44^, and^47^ we can see that the overall performance of our proposed work is better than the others.

**Table 2:** A comparison study between the proposed MTM sensor and others was reported in the literature.

References	Material sensing	Operating frequency (GHz)	Dielectric constant (DK) value	Shift of ${f}_{r}$ (MHz)	Amplitude change of ${S}_{11}$ (dB)	Q-factor	FOM
^20^	Transformer oil	2–6	2.7 for clean and 2.9 for waste transformer oil	40	− 1.5	60	34
^23^	Oil	1–8	2.74 for clean oil and 2.87 for waste oil	63	− 0.9	90	32
^27^	Diesel	8–12	2.72 for branded diesel and 2.48 for unbranded diesel	60	− 2.5	110	37
^28^	Diesel	8–12	2.73 for branded and 2.47 for unbranded diesel	120	− 3.2	105	41
^36^	Transformer oil	8–12	2.8 for clean and 2.7 for waste transformer oil	70	− 4.5	100	48
	Oil	8–12	2.78 for olive oil and 2.6 for corn oil	50	− 0.8		
^37^	Diesel	10–12	2.07 for branded and 2.68 for unbranded diesel	72	− 4	90	52
	Gasoline	10–11	2.43 for branded and 2.52 for unbranded gasoline	12	− 6		
^38^	Diesel	8–12	2.08 for pure and 2.7 for adulterated diesel	100	− 10	95	38
^44^	Gasoline	8–12	2.43 for branded and 2.51 for unbranded gasoline	28	− 8	105	43
	Diesel	8–12	2.07 for branded and 2.68 for unbranded diesel	92	− 20		
^47^	Transformer oil	8–12	2.9 for clean and 2.7 for waste transformer oil	70	− 3.7	75	36
	Lubricant	8–12	2.5 for clean and 2.3 for waste lubricant	60	− 4.1		
Proposed work	Olive and corn oils	8–12	2.56 for olive oil and 3.07 for corn oil	100	− 10.74	135	76
	Sunflower oil and palm oil	8–12	2.93 for sunflower oil and 2.81 for palm oil	80	− 5.99		
	Clean and waste brake oil	8–12	2.78 for clean and 2.69 for wase brake oil	70	− 3.97		
	Benzene and carbon-tetrachloride	8–12	2.55 for benzene and 2.51 for carbon-tetrachloride	90	− 3.62		

## Conclusion

Metamaterial sensor based on rectangular enclosed adjacent triple circle SRR shaped is presented for the recognition of various oils, fluids, and chemicals within the X band. The structure's performance has been studied both theoretically and empirically, and it has proven to be effective. This study was intended to provide a different approach to liquids sensing research. The whole dimension of the designed structure is 22.86 × 10.16 mm^2^, which is compatible with the X-band waveguide. The measured dielectric constant for olive oil is 2.56, and corn oil is 2.07, and the frequency has shifted 100 MHz with a reflection magnitude change of − 10.74 dB due to the variation of DK values between these two oils. The DK value for sunflower oil is 2.93, palm oil is 2.81, and the ${f}_{r}$ has shifted 80 MHz with a magnitude change of − 5.99 dB. The ${f}_{r}$ has shifted 70 MHz with a magnitude change of − 3.97 dB for clean and waste brake fluids, where the DK value is 2.78 for clean and 2.69 for waste brake fluids. The ${f}_{r}$ has shifted 90 MHz with an amplitude change of − 3.62 dB between benzene and carbon tetrachloride, where the DK value is 2.55 for benzene and 2.51 for carbon tetrachloride. The suggested MTM sensor has a good quality factor and high sensitivity in both frequency shifting and amplitude changing. The quality factor and FOM of the sensor are 135, and 76 which expresses its efficient performance. Furthermore, the proposed sensor can sensitively distinguish different liquids by using the frequency shifting property. For various oils, fluids, and chemicals, the measured and simulation findings are very similar. If the dielectric constant of the sample is changed, the obtained findings indicate that this idea can readily be applied to diverse applications of electrochemical sensing. Since the recommended sensor has high sensitivity, good-quality factor, excellent performance, and low price, hence it can be employed in a variety of applications, including industrial and liquid chemical detection.

## References

[1] Smith DR, Padilla WJ, Vier D, Nemat-Nasser SC, Schultz S (2000). Composite medium with simultaneously negative permeability and permittivity. Phys. Rev. Lett..

[2] Shelby RA, Smith DR, Schultz S (2001). Experimental verification of a negative index of refraction. Science.

[3] Withayachumnankul W, Jaruwongrungsee K, Fumeaux C, Abbott D (2011). Metamaterial-inspired multichannel thin-film sensor. IEEE Sens. J..

[4] Vafapour Z, Hajati Y, Hajati M, Ghahraloud H (2017). Graphene-based mid-infrared biosensor. JOSA B.

[5] Bakir M (2017). Electromagnetic-based microfluidic sensor applications. J. Electrochem. Soc..

[6] Xiong H, Long TB, Shi T, Jiang BX, Zhang JT (2020). Wideband and polarization-insensitive metamaterial absorber with loading lumped resistors. Appl. Opt..

[7] Islam MS, Samsuzzaman M, Beng GK, Misran N, Amin N, Islam MT (2020). A gap coupled hexagonal split ring resonator based metamaterial for S-band and X-band microwave applications. IEEE Access.

[8] Islam MR, Islam MT, Moniruzzaman M, Samsuzzaman M, Arshad H (2021). Penta band single negative meta-atom absorber designed on square enclosed star-shaped modified split ring resonator for S-, C-, X-and Ku-bands microwave applications. Sci. Rep..

[9] Johnson MC, Brunton SL, Kundtz NB, Kutz JN (2015). Sidelobe canceling for reconfigurable holographic metamaterial antenna. IEEE Trans. Antennas Propag..

[10] Nasimuddin N, Chen ZN, Qing X (2016). Bandwidth enhancement of a single-feed circularly polarized antenna using a metasurface: Metamaterial-based wideband CP rectangular microstrip antenna. IEEE Antennas Propag. Mag..

[11] Misran N, Yusop SH, Islam MT, Ismail MY (2012). Analysis of parameterization substrate thickness and permittivity for concentric split ring square reflectarray element. Jurnal Kejuruteraan (J. Eng.).

[12] Islam MR, Islam MT, Moniruzzaman M, Samsuzzaman M, Bais B, Arshad H (2020). Square enclosed circle split ring resonator enabled epsilon negative (ENG) near zero index (NZI) metamaterial for gain enhancement of multiband satellite and radar antenna applications. Results Phys..

[13] Bakir M, Karaaslan M, Altintas O, Bagmanci M, Akdogan V, Temurtas F (2018). Tunable energy harvesting on UHF bands especially for GSM frequencies. Int. J. Microw. Wirel. Technol..

[14] Bağmancı M, Karaaslan M, Unal E, Özaktürk M, Akgol O, Karadağ F (2019). Wide band fractal-based perfect energy absorber and power harvester. Int. J. RF Microwave Comput. Aided Eng..

[15] Chuma EL, Iano Y, Fontgalland G, Roger LLB (2018). Microwave sensor for liquid dielectric characterization based on metamaterial complementary split ring resonator. IEEE Sens. J..

[16] Hoque A, Islam MT, Almutairi AF, Chowdhury ME, Samsuzzaman M (2020). SNG and DNG meta-absorber with fractional absorption band for sensing application. Sci. Rep..

[17] Huang M, Yang J, Petrin A (2011). Microwave sensor using metamaterials. Wave Propagation.

[18] Islam MR, Islam MT, Hoque A, Soliman MS, Bais B, Sahar NM (2021). Tri circle split ring resonator shaped metamaterial with mathematical modelling for oil concentration sensing. IEEE Access.

[19] Islam MT, Hoque A, Almutairi AF, Amin N (2019). Left-handed metamaterial-inspired unit cell for S-Band glucose sensing application. Sensors.

[20] Altintaş O, Aksoy M, Ünal E, Karaaslan M (2019). Chemical liquid and transformer oil condition sensor based on metamaterial-inspired labyrinth resonator. J. Electrochem. Soc..

[21] Kim HK, Yoo M, Lim S (2015). Novel ethanol chemical sensor using microfluidic metamaterial. IEEE Int. Symp. Antennas Propagat. USNC/URSI Natl. Radio Sci. Meeting.

[22] Islam M, Islam MT, Soliman MS, Baharuddin MH, Mat K, Moubark AM (2021). Metamaterial based on an inverse double V loaded complementary square split ring resonator for radar and Wi-Fi applications. Sci. Rep..

[23] Abdulkarim YI, Deng L, Karaaslan M, Altıntaş O, Awl HN, Muhammadsharif FF (2020). Novel metamaterials-based hypersensitized liquid sensor integrating omega-shaped resonator with microstrip transmission line. Sensors.

[24] Patel SK, Surve J, Parmar J, Nguyen TK (2021). Review on graphene-based absorbers for infrared to ultraviolet frequencies. J. Adv. Eng. Computat..

[25] Parmar J, Patel SK (2022). Tunable and highly sensitive graphene-based biosensor with circle/split ring resonator metasurface for sensing hemoglobin/urine biomolecules. Physica B.

[26] Ahmed K, Haque MJ, Jabin MA, Paul BK, Amiri IS, Yupapin P (2019). Tetra-core surface plasmon resonance based biosensor for alcohol sensing. Physica B.

[27] Tamer A, Karadağ F, Ünal E, Abdulkarim YI, Deng L, Altintas O (2020). Metamaterial based sensor integrating transmission line for detection of branded and unbranded diesel fuel. Chem. Phys. Lett..

[28] Abdulkarim YI, Deng L, Karaaslan M, Dalgaç Ş, Mahmud RH, Alkurt FO (2020). The detection of chemical materials with a metamaterial-based sensor incorporating oval wing resonators. Electronics.

[29] Bakır M, Karaaslan M, Unal E, Karadag F, Alkurt FÖ, Altıntaş O (2018). Microfluidic and fuel adulteration sensing by using chiral metamaterial sensor. J. Electrochem. Soc..

[30] Soffiatti A, Max Y, Silva SG, de Mendonça LM (2018). Microwave metamaterial-based sensor for dielectric characterization of liquids. Sensors.

[31] Zaid J, Abdulhadi AE, Denidni TA (2019). Miniaturized multi-port microstrip patch antenna using metamaterial for passive UHF RFID-tag sensor applications. Sensors.

[32] J. Muñoz-Enano, P. Vélez, M. Gil, J. Mata-Contreras, K. Grenier, D. Dubuc*, et al.* Microstrip lines loaded with metamaterial-inspired resonators for microwave sensors/comparators with optimized sensitivity. in *2019 49th European Microwave Conference (EuMC)*, 2019, pp. 754–757.

[33] Awang RA, Tovar-Lopez FJ, Baum T, Sriram S, Rowe WS (2017). Meta-atom microfluidic sensor for measurement of dielectric properties of liquids. J. Appl. Phys..

[34] Abdolrazzaghi M, Katchinskiy N, Elezzabi A, Light PE, Daneshmand M (2021). Noninvasive glucose sensing in aqueous solutions using an active split-ring resonator. IEEE Sens. J..

[35] Kazemi N, Schofield K, Musilek P (2021). A high-resolution reflective microwave planar sensor for sensing of vanadium electrolyte. Sensors.

[36] Bakır M, Karaaslan M, Karadag F, Dalgac S, Ünal E, Akgöl O (2019). Metamaterial sensor for transformer oil, and microfluidics. Appl. Comput. Electromagnet. Soc. J..

[37] Tümkaya MA, Karaaslan M, Sabah C (2018). Metamaterial-based high efficiency portable sensor application for determining branded and unbranded fuel oil. Bull. Mater. Sci..

[38] Tamer A, Alkurt FO, Altintas O, Karaaslan M, Unal E, Akgol O (2018). Transmission line integrated metamaterial based liquid sensor. J. Electrochem. Soc..

[39] Lee Y, Kim S-J, Park H, Lee B (2017). Metamaterials and metasurfaces for sensor applications. Sensors.

[40] Vélez P, Su L, Grenier K, Mata-Contreras J, Dubuc D, Martín F (2017). Microwave microfluidic sensor based on a microstrip splitter/combiner configuration and split ring resonators (SRRs) for dielectric characterization of liquids. IEEE Sens. J..

[41] Bahar AAM, Zakaria Z, Arshad MM, Isa A, Dasril Y, Alahnomi RA (2019). Real time microwave biochemical sensor based on circular SIW approach for aqueous dielectric detection. Sci. Rep..

[42] Lee H-J, Yook J-G (2008). Biosensing using split-ring resonators at microwave regime. Appl. Phys. Lett..

[43] Tümkaya MA, Dinçer F, Karaaslan M, Sabah C (2017). Sensitive metamaterial sensor for distinction of authentic and inauthentic fuel samples. J. Electron. Mater..

[44] Tümkaya MA, Ünal E, Sabah C (2019). Metamaterial-based fuel sensor application with three rhombus slots. Int. J. Mod. Phys. B.

[45] Islam MT, Islam MR, Islam MT, Hoque A, Samsuzzaman M (2020). Linear regression of sensitivity for meander line parasitic resonator based on ENG metamaterial in the application of sensing. J. Mater. Res. Technol..

[46] Abdulkarim YI, Deng L, Karaaslan M, Unal E (2019). Determination of the liquid chemicals depending on the electrical characteristics by using metamaterial absorber based sensor. Chem. Phys. Lett..

[47] Altıntaş O, Aksoy M, Ünal E (2020). Design of a metamaterial inspired omega shaped resonator based sensor for industrial implementations. Physica E Low-dimensional Syst. Nanostruct..

[48] Abdulkarim YI, Deng L, Luo H, Huang S, Karaaslan M, Altıntaş O (2020). Design and study of a metamaterial based sensor for the application of liquid chemicals detection. J. Market. Res..

[49] Abdulkarim YI, Dalgaç Ş, Alkurt FO, Muhammadsharif FF, Awl HN, Saeed SR (2021). Utilization of a triple hexagonal split ring resonator (SRR) based metamaterial sensor for the improved detection of fuel adulteration. J. Mater. Sci.: Mater. Electron..

[50] CST AG, D. CST Studio Suite, www.3ds.com/products-services/simulia/products/cst-studio-suite (2019).

[51] Saha C, Siddiqui JY, Antar YM (2011). "Square split ring resonator backed coplanar waveguide for filter applications. XXXth URSI General Assembly Sci. Symp..

[52] Abdolrazzaghi M, Daneshmand M, Iyer AK (2018). Strongly enhanced sensitivity in planar microwave sensors based on metamaterial coupling. IEEE Trans. Microw. Theory Tech..

[53] Omer AE, Shaker G, Safavi-Naeini S, Kokabi H, Alquié G, Deshours F (2020). Low-cost portable microwave sensor for non-invasive monitoring of blood glucose level: Novel design utilizing a four-cell CSRR hexagonal configuration. Sci. Rep..

[54] Shaw T, Mitra D (2017). Electromagnetic metamaterial based sensor design for chemical discrimination. IEEE MTT-S Int. Microwave RF Conf. (IMaRC).

[55] Cong L, Tan S, Yahiaoui R, Yan F, Zhang W, Singh R (2015). Experimental demonstration of ultrasensitive sensing with terahertz metamaterial absorbers: A comparison with the metasurfaces. Appl. Phys. Lett..

[56] Lizhi H, Toyoda K, Ihara I (2008). Dielectric properties of edible oils and fatty acids as a function of frequency, temperature, moisture and composition. J. Food Eng..

