# Author Correction: Metamaterial sensor based on rectangular enclosed adjacent triple circle split ring resonator with good quality factor for microwave sensing application

**DOI:** 10.1038/s41598-024-60569-7

**Published:** 2024-04-29

**Authors:** Md. Rashedul Islam, Mohammad Tariqul Islam, M. Salaheldeen M., Badariah Bais, Sami H. A. Almalki, Haitham Alsaif, Md. Shabiul Islam

**Affiliations:** 1 Department of Electrical, Electronic and Systems Engineering, Faculty of Engineering and Built Environment, Universiti Kebangsaan Malaysia, 43600 Bangi, Selangor Malaysia; 2 Department of Electrical Engineering, Faculty of Energy Engineering, Aswan University, Aswan, 81528 Egypt; 3 Department of Electrical Engineering, College of Engineering, Taif University, P.O. Box 11099, Taif, 21944 Kingdom of Saudi Arabia; 4 Department of Electrical Engineering, College of Engineering, University of Ha’il, Ha’il 81481, Saudi Arabia; 5 Faculty of Engineering, Multimedia University, 63100 Cyberjaya, Selangor Malaysia

Correction to: *Scientific Reports* 10.1038/s41598-022-10729-4, published online 26 April 2022

The original version of this Article contained an error in the Acknowledgements section.

“This work was supported by the Ministry of Higher Education of Malaysia, Research Grant code: FRGS/1/2021/TK0/UKM/02/18. This work was also supported by Taif University Researchers Supporting Project number (TURSP-2020/206), Taif University, Taif, Kingdom of Saudi Arabia.”

now reads,

“This research was funded by the Ministry of Higher Education (MOHE) through the Fundamental Research Grant Scheme (FRGS) under the grant number FRGS/1/2021/TK0/UKM/02/18. This work was also supported by Taif University Researchers Supporting Project number (TURSP-2020/206), Taif University, Taif, Kingdom of Saudi Arabia.”

The original Article has been corrected.

